# Transcriptome and microbiome-immune changes across preinvasive and invasive anal cancer lesions

**DOI:** 10.1172/jci.insight.180907

**Published:** 2024-07-18

**Authors:** Ezequiel Lacunza, Valeria Fink, María E. Salas, Ana M. Gun, Jorge A. Basiletti, María A. Picconi, Mariano Golubicki, Juan Robbio, Mirta Kujaruk, Soledad Iseas, Sion Williams, María I. Figueroa, Omar Coso, Pedro Cahn, Juan C. Ramos, Martín C. Abba

**Affiliations:** 1 Centro de Investigaciones Inmunológicas Básicas y Aplicadas (CINIBA), Facultad de Ciencias Médicas, Universidad Nacional de La Plata, La Plata, Argentina.; 2 University of Miami - Center for AIDS Research/Sylvester Cancer Comprehensive Center Argentina Consortium for Research and Training in Virally Induced AIDS-Malignancies, University of Miami Miller School of Medicine, Miami, Florida, USA (detailed in Supplemental Acknowledgments).; 3 Dirección de Investigaciones, Fundación Huésped, Buenos Aires, Argentina.; 4 Laboratorio Nacional y Regional de Referencia de Virus Papiloma Humano, Instituto Nacional de Enfermedades Infecciosas - ANLIS “Dr. Malbrán”, Buenos Aires, Argentina.; 5 Unidad de Oncología, Hospital de Gastroenterología “Dr. Carlos Bonorino Udaondo”, Buenos Aires, Argentina.; 6 Medical Oncology Department, Paris-St Joseph Hospital, Paris, France.; 7 University of Miami - Center for AIDS Research/Sylvester Comprehensive Cancer Center, University of Miami Miller School of Medicine, Miami, Florida, USA.; 8 Instituto de Fisiología, Biología Molecular y Neurociencias (IFIBYNE-CONICET), Universidad de Buenos Aires, Buenos Aires, Argentina.

**Keywords:** Cell biology, Oncology, Bioinformatics, Expression profiling, Molecular biology

## Abstract

Anal squamous cell carcinoma (ASCC) is a rare gastrointestinal malignancy linked to high-risk human papillomavirus (HPV) infection, which develops from precursor lesions like low-grade squamous intraepithelial lesions and high-grade squamous intraepithelial lesions (HGSILs). ASCC incidence varies across populations and poses increased risk for people living with HIV. Our investigation focused on transcriptomic and metatranscriptomic changes from squamous intraepithelial lesions to ASCC. Metatranscriptomic analysis highlighted specific bacterial species (e.g., *Fusobacterium nucleatum*, *Bacteroides fragilis*) more prevalent in ASCC than precancerous lesions. These species correlated with gene-encoding enzymes (Acca, glyQ, eno, pgk, por) and oncoproteins (FadA, dnaK), presenting potential diagnostic or treatment markers. Unsupervised transcriptomic analysis identified distinct sample clusters reflecting histological diagnosis, immune infiltrate, HIV/HPV status, and pathway activities, recapitulating anal cancer progression’s natural history. Our study unveiled molecular mechanisms in anal cancer progression, aiding in stratifying HGSIL cases based on low or high risk of progression to malignancy.

## Abstract 2

Anal Squamous Cell carcinoma (ASCC) is a rare malignancy often preceded by squamous intraepithelial lesions. Our study delineates transcriptomic and microbiome-immune shifts in ASCC progression.

## Introduction

Anal squamous cell carcinoma (ASCC), a rare gastrointestinal neoplasia, involves the formation of malignant tumors in the anal region. Over the past 30 years, ASCC incidence has risen globally, particularly in men who have sex with men (MSM) and people living with HIV (PLWH) (1).

Squamous intraepithelial lesions (SILs), categorized into low-grade SILs (LGSILs), analogous to anal intraepithelial neoplasia I, and high-grade SILs (HGSILs), analogous to anal intraepithelial neoplasia II and III, often precede progression to ASCC (2, 3). Similar to cervical cancer, ASCC development is driven by infection with oncogenic human papillomaviruses (HPVs) (4, 5). The risk of anal cancer varies significantly across different population groups, with the highest risk observed in PLWH (1). This increased susceptibility is primarily attributed to a weakened immune system, which makes it more challenging to control infections, including HPV infections (6). Beyond the potential impact of oncogenic viruses, the microbiome may also play a significant role in the development of precancerous anal lesions and ASCC, as the influence of microbes is increasingly recognized in cancer development (7, 8). The microbiome can influence the balance of host cell proliferation and apoptosis, disrupt antitumor immunity, and affect the metabolism of host-produced factors, ingested food components, and drugs (9). In a recent study, we defined the microbiome composition of the anal mucosa of HIV-exposed individuals. Metagenomic sequencing enabled us to identify viral and bacterial taxa linked to the development of anal lesions. Our results verified the occurrence of oncogenic viromes in this population and identified *Prevotella bivia* and *Fusobacterium gonidiaformans* as 2 relevant bacterial species predisposing to SILs. Moreover, gene family analysis identified bacterial gene signatures associated with SILs that may have potential as prognostic and predictive biomarkers for HIV-associated malignancies (10). Other reports using 16S rRNA gene sequencing to analyze the ASCC demonstrated the role of the anal microbiota in anal cancer response to therapy and toxicity, as well as changes in taxonomic compositions among normal, dysplasia, and anal cancer samples (11, 12).

The molecular biology of ASCC is complex and not completely understood (13). However, studies have identified potential molecular targets for ASCC therapy, including regulators of apoptosis (14), agents targeting the PI3K/AKT pathway (15), and antibody therapy targeting EGFR (16) or programmed cell death ligand 1 (PD-L1) expression to stratify good versus poor responders to chemoradiotherapy (17). Despite advancements in understanding ASCC from various perspectives, thus far, no prognostic or predictive markers have been identified that are useful in clinical practice. Furthermore, a notable gap in existing information is the paucity of studies using anal cancer biopsies for gene expression profiling, particularly utilizing advanced techniques like next-generation sequencing (NGS).

Transcriptomic and metatranscriptomic profiling are powerful NGS-based tools for the functional genomics characterization of complex diseases. In this sense, bulk RNA sequencing (RNA-Seq) in neoplastic disease enables simultaneous study of the host tumor transcriptome and its microenvironment, including the tumor immune infiltrate and associated tumor microbiome. Transcriptomic profiling provides thorough examination of gene expression patterns, uncovering crucial insights into the molecular mechanisms driving cancer development and progression. Metatranscriptomic profiling enables researchers to analyze gene expression levels of various organisms within a microbial community, providing insights into their metabolic processes and functional activities in cancer and immune-related diseases (18). In this sense, metatranscriptomic approaches enable the analysis of the active microbiota instead of more frequent studies based on 16S rRNA sequencing, which analyzes the “total” microbiota, including active and inactive bacteria.

The aim of this study was to analyze the transcriptomic and metatranscriptomic changes during the progression from LGSIL to HGSIL and ultimately to ASCC.

We collected biopsies identified as SILs and ASCC from a cohort of 70 participants, both with and without HIV, who provided informed consent. Biopsies were subjected to bulk RNA-Seq. Our goal was to gain insights into the molecular mechanisms underlying the development and progression of anal lesions, which could lead to identifying novel biomarkers and therapeutic targets for improved diagnostic and treatment strategies in patients with ASCC.

## Results

### Clinical characteristics of patients and microbial community variations in SILs and ASCC cases.

Seventy patients were included in the study. All underwent anal cytology and high-resolution anoscopy with biopsies. Based on cytology and histology analysis, samples were classified into LGSIL, *n* = 23; HGSIL, *n* = 16; and ASCC, *n* = 23. Demographic and clinical data were collected, including age, sex at birth (male or female), gender (cisgender men, CGM; transgender women, TGW; and cisgender women, CGW), HPV DNA status, HIV status, and antiretroviral therapy (ART). This information is summarized in Table 1.

**Figure 1:**
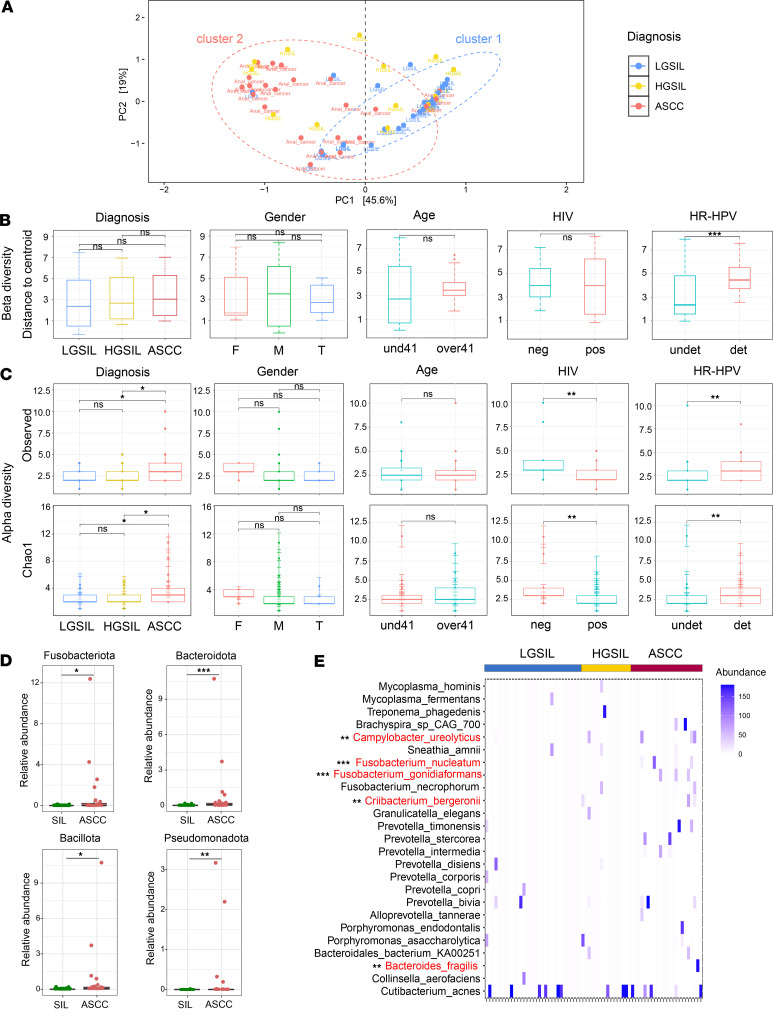
Richness, diversity, and microbial profile of LGSIL, HGSIL, and ASCC. (**A**) Principal coordinate analysis depicting the unsupervised distribution of samples, assessed at the species level based on microbiota composition and evaluated through Euclidean distance. (**B**) β-Diversity comparison between diagnosis groups and covariates. (**C**) Observed and Chao1 richness indices obtained at species level by metatranscriptome analysis. (**D**) Significantly altered phyla Fusobacteriota, Bacteroidota, Bacillota, and Pseudomonadota were related to ASCC. Statistical significance was calculated with Wilcoxon’s signed-rank test. (**E**) Heatmap representation of the relative abundances of the most abundant bacterial species identified across all samples. Highlighted in red are the taxa significantly enriched in ASCC compared with SIL obtained by MaAsLin2 analysis. *P < 0.05; **P < 0.01; ***P < 0.001.

We first conducted a compositional analysis of the 3 distinct groups — LGSIL, HGSIL, and ASCC — by performing permutational multivariate ANOVA (PERMANOVA) with Euclidean distance. The principal coordinate analysis (PCoA) defined 2 distinct clusters based on component I (*P* < 0.001). Cluster I was enriched in LGSILs, comprising 24 out of 31 samples (77%), while cluster II predominantly featured ASCC samples with 19 out of 23 (83%) (Figure 1A). HGSIL demonstrated an almost equal distribution between the 2 clusters, with 9 out of 16 in cluster I (56%) and 7 out of 16 in cluster II (44%) (Figure 1A). In addition, we considered covariates such as age, gender, HIV status, and high-risk HPV (HR-HPV) DNA genotyping to evaluate the factors influencing cluster formation based on diagnostic groups. Using PERMANOVA, our analysis of β-diversity revealed distinctions primarily in samples positive for HR-HPV types compared with samples in which these HPV types were undetectable (Figure 1B and Supplemental Figure 1; supplemental material available online with this article; https://doi.org/10.1172/jci.insight.180907DS1).

The ASCC microbial community, assessed through Observed and Chao 1 indices based on metatranscriptome species composition, exhibited a significantly higher richness compared with LGSIL (Observed, *P* = 0.033; Chao 1, *P* = 0.035) and HGSIL (Observed, *P* = 0.029; Chao 1, *P* = 0.034). This trend persisted when merging LGSIL and HGSIL into the group termed SILs (Observed, *P* = 0.012; Chao 1, *P* = 0.018), suggesting the ASCC environment provides a more favorable habitat for a specific range of microorganisms, resulting in increased community richness (Figure 1B and Supplemental Data 1). Richness indices were also augmented in the HR-HPV group compared with the HR-HPV^–^ cohort. In addition, a significant association between HIV-positive status and decreased α-diversity was observed, in agreement with previous studies (10) (Figure 1C).

Analysis of diversity indices (Shannon and Simpson) revealed a significant increase in ASCC compared with HGSIL (Shannon, *P* = 0.0082; Simpson, *P* = 0.0134), while no differences were observed between LGSIL and ASCC (Supplemental Data 1). These findings align with a recent study reporting similar α-diversity indices between anal dysplasia and anal cancer but elevated abundance of specific taxa in the latter (12). Consistent with our prior research, we further observed a negative influence of aging on microbiome diversity (10) (Supplemental Data 1).

We analyzed phylum-level bacterial abundance between SILs and ASCC groups. Fusobacteriota, Bacteroidota, and Bacillota, among the most abundant phyla, were significantly more enriched in ASCC than SILs (Figure 1D). Additionally, Pseudomonadota showed enrichment within the ASCC group compared with precancerous lesions (Figure 1D).

At the species level, we identified a total of 25 taxa, each exhibiting a relative abundance exceeding 20% of the overall composition in at least 1 of the samples (Figure 1E). Among these taxa, *F*. *nucleatum*, *F*. *necrophorum*, *B*. *fragilis*, and *P*. *intermedia* are well-established gut-associated bacteria with previous associations with colorectal cancer (CRC) (19). Conversely, other taxa such as *M*. *hominis*, *P*. *bivia*, *F*. *gonidiaformans*, *S*. *amnii*, *C*. *ureoliticus*, or *B*. *fragilis* have been linked to HPV-related precancerous and cancerous genital lesions (10, 12, 20–22).

To identify bacterial species associated with ASCC compared with SILs, we used MaAsLin2 analysis. To account for potential confounders, we refined the model by incorporating additional covariates, including HIV status, HR-HPV DNA status, sex at birth, and age. Significant enrichment was observed for *F*. *nucleatum* (*P* = 0.001), *F*. *gonidiaformans* (*P* = 0.001), *B*. *fragilis* (*P* = 0.01), *C*. *ureolyticus* (*P* = 0.003), and *C*. *bergeronii* (*P* = 0.006) (Figure 1E and Supplemental Data 2). Moreover, *C*. *ureolyticus* (*P* = 0.002), *F*. *gonidiaformans* (*P* = 0.01), and *C*. *bergeronii* (*P* = 0.02) were associated with male sex (Supplemental Data 2). Additionally, *C*. *ureolyticus* correlated with HIV-negative cases (*P* = 0.03) (Supplemental Data 2).

*F. nucleatum* and *B*. *fragilis* have established roles in CRC progression, highlighting their importance in ASCC development and progression (19). While knowledge about *F*. *gonidiaformans*, *C*. *ureolyticus*, and *C*. *bergeronii* is limited, prior associations exist between *F*. *gonidiaformans* and *C*. *ureolyticus* with HPV presence and the development of precancerous lesions in anal and cervical cancers (10, 21, 23). These findings suggest a potential contribution of specific bacteria to ASCC progression.

### Exploring viral signatures in anal lesion progression: A. papillomavirus and non-HPV species.

**Figure 2:**
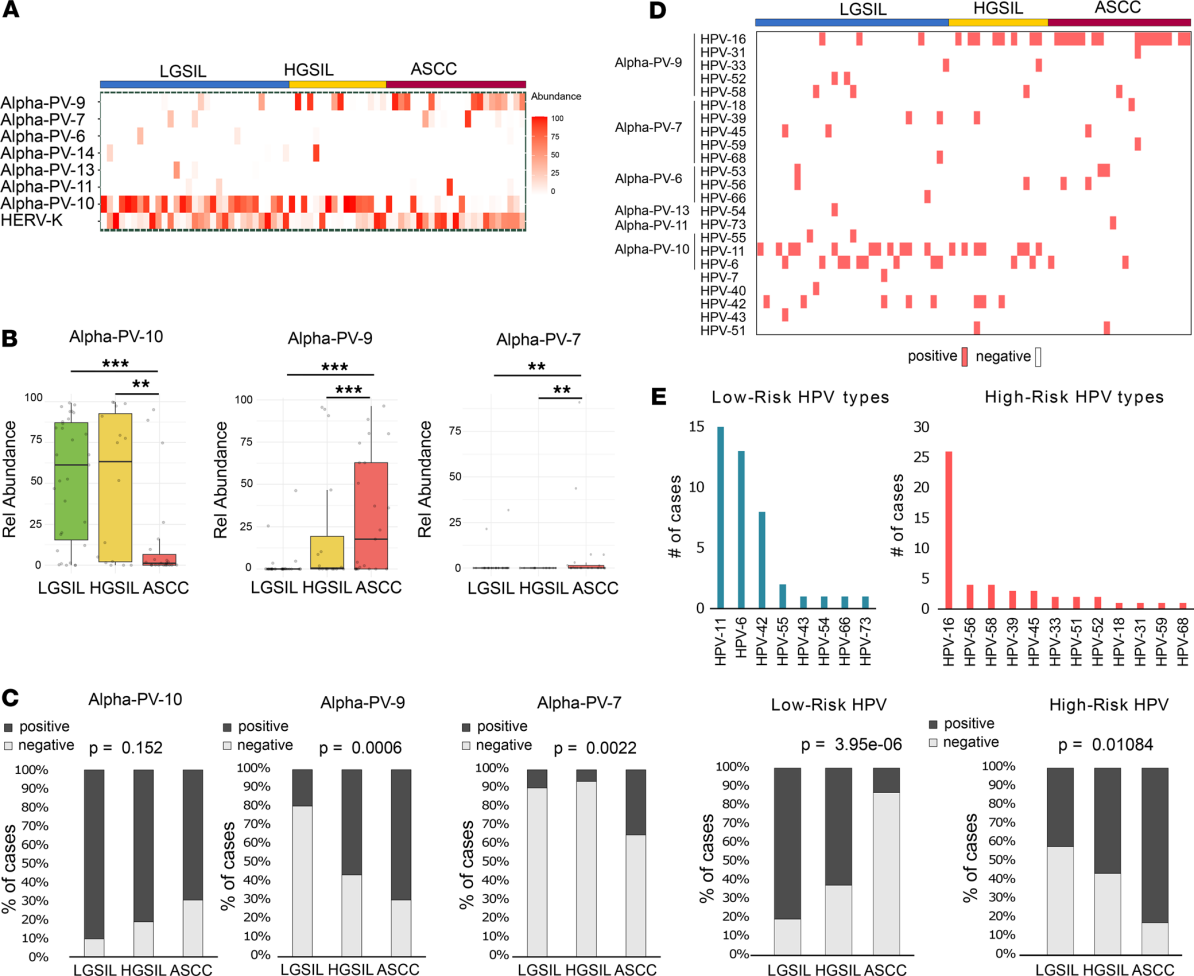
Viral composition of LGSIL, HGSIL, and ASCC. (**A**) Relative abundance heatmap showing the most prevalent viral species identified in the diagnosis groups using metatranscriptome analysis (RNA level). (**B**) Alpha-PV-10 was found to be linked to the SIL group, whereas Alpha-PV-9 and Alpha-PV-7 were associated with ASCC. Statistical significance was derived from MaAsLin2 analysis. (**C**) Percentage of patients with detectable viruses of the species Alpha-PV-10, -9, and -7 assessed by metatranscriptome analysis. (**D**) Heatmpa visualizing the HPV types identified through DNA genotyping across the different diagnosis groups. (**E**) Percentage distribution of HPV types, assessed by DNA genotyping and classified into low-risk (LR) and high-risk (HR) categories. (**F**) Percentage of patients in each diagnostic group with detectable LR- and HR-HPV types identified through DNA genotyping. Statistical significance was determined through application of Fisher’s exact test. **P < 0.01; ***P < 0.001.

In terms of viral composition analysis, among the 40 species identified at the transcript level in all samples, 8 were the most prevalent, with abundances greater than 30% of the total abundance in any sample and detected more than 3 times. Notably, 7 of these species belonged to the *A*. *papillomavirus* (Alpha-PV) genus, along with the human endogenous retrovirus K (HERV-K), with evident variations in their relative abundances across distinct diagnostic groups (Figure 2A). MaAsLin2 analysis revealed a higher abundance of Alpha-PV-10, which includes low-risk (LR) genotypes like HPV6 and HPV11, in both LGSIL and HGSIL compared with ASCC (Figure 2, A and B, and Supplemental Data 2). Conversely, Alpha-PV-9 (HPV16, 31, 33, 52, 58) and Alpha-PV-7 (HPV18, 39, 59, 68, 45, 70) were significantly associated with HGSIL and ASCC (Figure 2, A and B, and Supplemental Data 2). This trend persisted when considering the number of positive cases for these species independent of their relative abundance (Figure 2C). Although the significance was not established for Alpha-PV-10, it remained significant for Alpha-PV-7 and Alpha-PV-9 (Figure 2C).

The HPV DNA genotyping data highlighted a robust association between HPV16 and both HGSIL and ASCC, correlating with the pattern observed with Alpha-PV-9 (Figure 2C). However, HPV18 was detected in only 1 case of ASCC, contrasting with Alpha-PV-7 detected at the RNA level in over 20% of participants (Figure 2A). This discrepancy could be due to Alpha-PV-7 containing other HPV genotypes (24). HPV6 and HPV11 were predominantly linked to LGSIL (Figure 2D). Analyzing positive and negative cases for all LR-HPV types and HR-HPV types identified within the cohort revealed negative (*P* < 0.001) and positive associations (*P* < 0.05), respectively, with the diagnostic groups (Figure 2, E and F). These results verify the prominence of HR- and LR-HPV types, particularly HPV6 and HPV16, in delineating the diagnostic groups (25).

Among the non-HPV species, we highlight a significant increase in the relative abundance of the endogenous HERV-K in ASCC compared with HGSIL (*P* < 0.01; Supplemental Data 2). HERV-K overexpression is widely associated with malignant phenotypes and is upregulated in various cancers such as breast lymphoma, germ-line tumors, and melanoma (26). Additionally, human betaherpesvirus 5 (HCMV), although with low relative abundance, demonstrated significant enrichment in ASCC compared with SILs (*P* < 0.05; Supplemental Data 2). HCMV is linked to several cancer types, including lymphoma, cervical cancer, Kaposi’s sarcoma, CRC, prostate cancer, skin cancer, and glioblastomas (27). However, it remains unclear whether HCMV actively contributes to malignant tumor progression or is reactivated under conditions leading to chronic inflammation or immunosuppression (27).

Overall, these findings verify the significance of specific viral Alpha-PV species and their association with SILs toward ASCC progression. Furthermore, our data reveal a potential involvement of HERV-K and HCMV in ASCC tumorigenesis. Additionally, metatranscriptomics demonstrates reliability, sensitivity, and specificity in detecting the presence of HPV types, even in cases where DNA genotyping results were negative.

### Metabolic pathways in ASCC progression.

To understand the functional implications of microbial community changes between SILs and ASCC, we conducted metatranscriptomics, revealing 20 MetaCyc modules as significantly enriched pathways in ASCC compared with SILs (Table 2). These modules encompassed nucleotide, amino acid, and lipid biosynthesis pathways. This finding aligns with our prior observations, where pathways related to amino acid and de novo nucleotide biosynthesis were enriched in HIV-postive individuals with anal precancerous lesions (10). These pathways are vital for cell growth and proliferation, as cells require energy and nutrients from their environment to support these processes. Similarly, cancer cells exhibit metabolic adaptations essential for their growth (28). Hence, our data suggest that certain bacteria within the evolving microenvironment during malignancy may exploit these pathways to thrive and proliferate, like cancer cells.

### Microbial contributions to anal lesions: enriched proteins and taxonomic associations.

**Figure 3:**
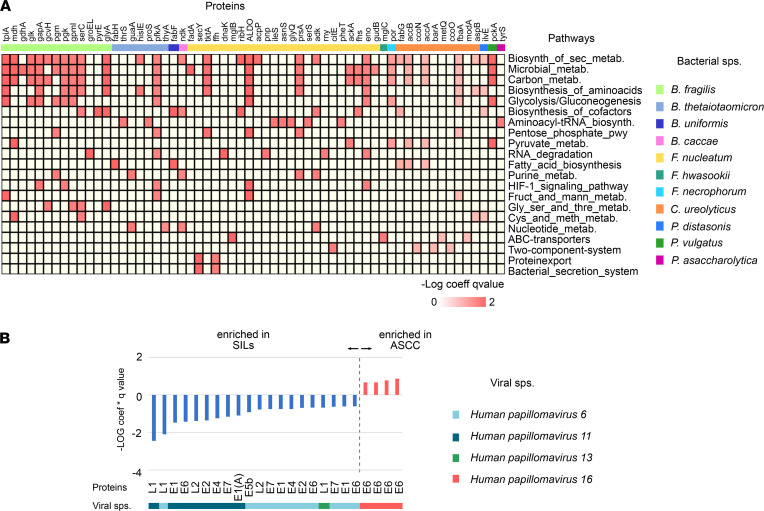
Functional and taxonomic enrichment of microbial gene proteins associated with anal lesions. (**A**) Heatmap representation of metabolic pathways enriched in ASCC compared with SILs represented by 60 gene proteins contributed by relevant gut microbiota taxa, of which *F*. *nucleatum*, *B*. *fragilis*, and *C*. *ureolyticus* are predominant. (**B**) Viral proteins identified as differentially abundant in ASCC relative to SILs contributed by high-risk and low-risk HPV.

To go further, we next explored the gene proteins contributed by the microbial organisms in the comparison of SILs versus ASCC. MaAsLin2 analysis yielded 2,523 UniRef90 sequence proteins differentially expressed (Supplemental Data 3). We further employed the Kyoto Encyclopedia of Genes and Genomes (KEGG) database to annotate 387 proteins, of which 349 were significantly enriched in ASCC and 37 in SILs (Supplemental Data 3). Functional annotation using KEGG Mapper revealed metabolic pathways such as glycolysis, lipid, amino acid, and nucleotide biosynthesis, contributed by 60 bacterial proteins enriched in ASCC (Figure 3A). Proteins like Acca (acetyl-CoA carboxylase carboxyl transferase subunit alpha), glyA (glycine hydroxymethyltransferase), glyQ (glycyl-tRNA synthetase alpha chain), eno (enolase), pgk (phosphoglycerate kinase), and por (pyruvate-ferredoxin/flavodoxin oxidoreductase) were previously identified in anal samples from individuals with precancerous anal lesions (10), underlining their potential roles as metabolic markers in anal cancer progression. In addition, among these 60 proteins, we identified the enrichment of the oncogenic FadA adhesion protein from *F*. *nucleatum* in ASCC, a factor widely associated with CRC; and dnaK, a protein kinase with a known involvement in carcinogenesis and cancer progression (29, 30). These findings align with the taxonomic abundance analysis, highlighting the significant role of bacteria like *B*. *fragilis*, *F*. *nucleatum*, and *C*. *ureolyticus*, alongside other relevant and distinct gut microbiota taxa, in orchestrating these processes (Figure 3A). Furthermore, 4 proteins linked to the oncogene E6 from HPV16 were enriched in ASCC (Figure 3B). E6 oncoprotein promotes p53 degradation, contributing to keratinocyte immortalization. In SILs, 37 enriched proteins were detected, all predominantly associated with genes from the LR-HPV genomes HPV6 and HPV11, underscoring their potential role as drivers or sustainers of precancerous anal lesions (31) (Figure 3B).

### Transcriptomic profiling and functional insights across anal lesion progression.

**Figure 4:**
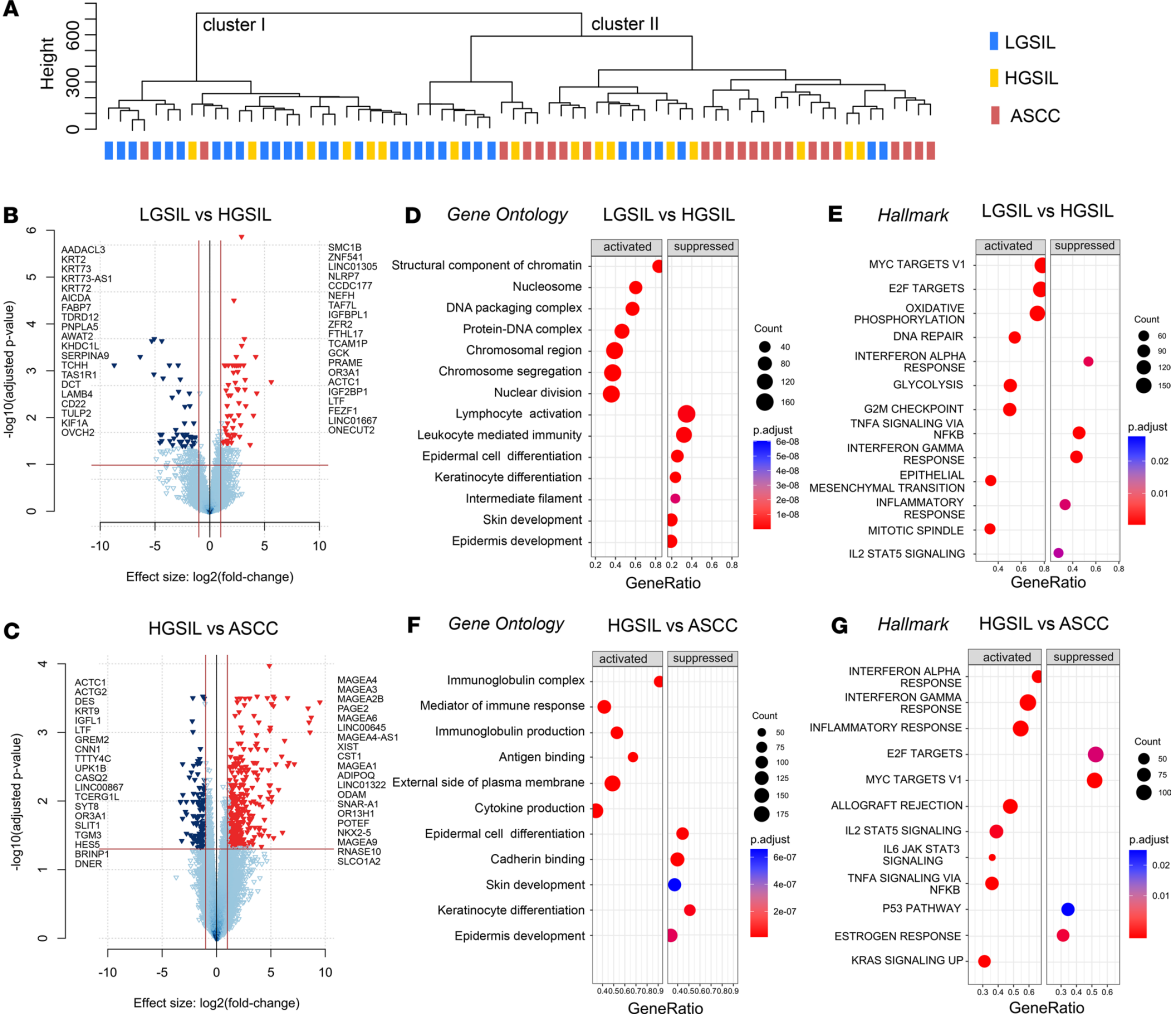
Differential gene expression analysis and functional enrichment of transcriptomic data. (**A**) Unsupervised hierarchical clustering of samples classified according to diagnosis groups. (**B** and **C**) Volcano plots representing significant DEGs (logFC > 1, adj *P* < 0.05) from the comparisons between LGSIL and HGSIL (**B**) and between HGSIL and ASCC (**C**). Upregulated genes are indicated by red arrowheads, while downregulated genes are indicated by blue arrowheads. The top 20 significant genes are shown. (**D**–**G**) Dot plots of GSEA obtained from the comparisons between LGSIL and HGSIL (**D** and **E**) and between HGSIL and ASCC (**F** and **G**). (**D**) Dot plot of significantly activated and suppressed GO pathways in HGSIL compared with LGSIL. (**E**) Dot plot of significantly activated and suppressed Hallmarks of Cancer in HGSIL compared with LGSIL. (**F**) Dot plot of significantly activated and suppressed GO pathways in ASCC compared with HGSIL. (**G**) Dot plot of significantly activated and suppressed Hallmarks of Cancer in ASCC compared with HGSIL.

We then explored the host transcriptome of LGSIL, HGSIL, and ASCC. Like the metatranscriptome analysis, the unsupervised clustering of samples revealed 2 primary clusters (Figure 4A). Cluster I was predominantly composed of LGSILs, with the inclusion of some HGSILs. In contrast, cluster II comprised mostly anal cancer samples, alongside a subgroup of SILs. One plausible interpretation for this distribution is that precancerous lesions may be at varying stages of progression, with some nearing malignant transformation and others in a regressive or early stage (32).

Next, we applied supervised comparative analysis between LGSIL and HGSIL as well as HGSIL and ASCC. The analysis revealed a higher number of differentially expressed genes (DEGs; fold-change [FC] > 2, FDR < 0.05) in the transition from HGSIL to ASCC (544 DEGs) than in the comparison among the 2 SIL groups (121 DEGs) (Figure 4, B and C, and Supplemental Data 4). Among the most significant genes, a decrease in keratins in HGSIL compared with LGSIL stands out (Figure 4B) as well as the overexpression of members of the MAGE gene family of cancer/testis antigens in ASCC compared with HGSIL, like *MAGEA4*, *MAGEA3*, and *MAGEA1* (Figure 4C). The MAGE family has gained attention as a potential cancer biomarker and immunotherapy (33). Notably, a phase I trial for autologous T cell therapy targeting *MAGEA4*-positive solid cancers is currently underway (34).

To comprehend the functional significance of DEGs, we used gene set enrichment analysis (GSEA) on Gene Ontology (GO), Cancer Hallmarks, and Disease Ontology (DO) terms. GSEA revealed activated processes such as nuclear division, chromatin modification, and cell proliferation, along with suppressed pathways like keratinocyte differentiation and leukocyte-mediated immunity in HGSIL compared with LGSIL (Figure 4D and Supplemental Data 5). These processes align with the histopathological features of HGSIL, including a higher nuclear-to-cytoplasmic ratio, decreased organization of cell layers, a greater degree of nuclear pleomorphism, and increased mitotic index (35). Furthermore, analysis of Cancer Hallmarks indicated the activation of pathway terms associated with sustaining proliferative signaling, such as MYC targets, E2F targets, G2M checkpoint, or mitotic spindle (Figure 4E). Notably, there was a decrease in genes related to IFN-α and IFN-γ levels, potentially compromising the ability of the immune system to mount an effective defense against viral infections and favoring persistent infection and progression to HGSIL (36) (Figure 4E). The activation of DNA repair genes may be a response to potential damage caused by viral oncoproteins E6 or E7, which aim to integrate the host genome through DNA double breakpoints (35) (Figure 4E).

The network representation resulting from GSEA with GO comparing LGSIL to HGSIL provided valuable insights into the molecular landscape (Supplemental Figure 2A). Three distinct clusters emerged, each revealing specific functional themes: a DNA and chromosome organization cluster, characterized by a dense interconnection of genes primarily related to histones and chromatin modifiers, suggesting a potential role in the epigenetic regulation and structural integrity of the genome; a chromosome segregation cluster with genes predominantly linked to processes such as the mitotic spindle and cell division; and a skin development cluster, offering insights into the gene network governing epidermal differentiation (Supplemental Figure 2A). These findings suggest a complex interplay of molecular events involving DNA organization, chromosome segregation, and skin differentiation in the transition from LGSIL to HGSIL. Some of these events may be attributed to HPV E6 oncoprotein. The expression of viral E6 enhances cell cycle progression and induces mitotic defects leading to centrosome amplification observed in keratinocytes, contributing to chromosomal instability through aberrant chromosome segregation (37).

Moreover, DO revealed additional clusters of genes related to gut inflammatory processes, HIV disease, and B cell immunodeficiency (Supplemental Figure 2B). Together, these data unveil the impact on the anal transcriptome caused during transition from LGSIL to HGSIL, defining distinct driver processes, including several genes that can be new avenues for further research.

Conversely, in comparing HGSIL and ASCC, GO analysis revealed a predominant activation of immune response in ASCC but a decrease in epidermal differentiation-related genes (Figure 4F). Hallmarks analysis demonstrated activation of IFN pathways emphasizing immune activation. Remarkably, suppression of the p53 pathway may be linked to the overexpression of HPV16 E6 protein (Figure 4G). The network representation of GO revealed clusters of genes mainly representing immune activation, leukocyte migration, and cytokine and immunoglobulin production but also epidermal cell differentiation (Supplemental Figure 2C). Additionally, DO yielded terms related to inflammatory processes of colon, HIV, and skin disease (Supplemental Figure 2D).

Therefore, unlike the comparison between LGSIL and HGSIL, the data suggest that the transition from HGSIL to ASCC is characterized by a predominance of immune response activation over processes related to cell proliferation or DNA modifications (38).

### Host transcriptome reveals 2 intrinsic signatures with varied features and prognoses.

**Figure 5:**
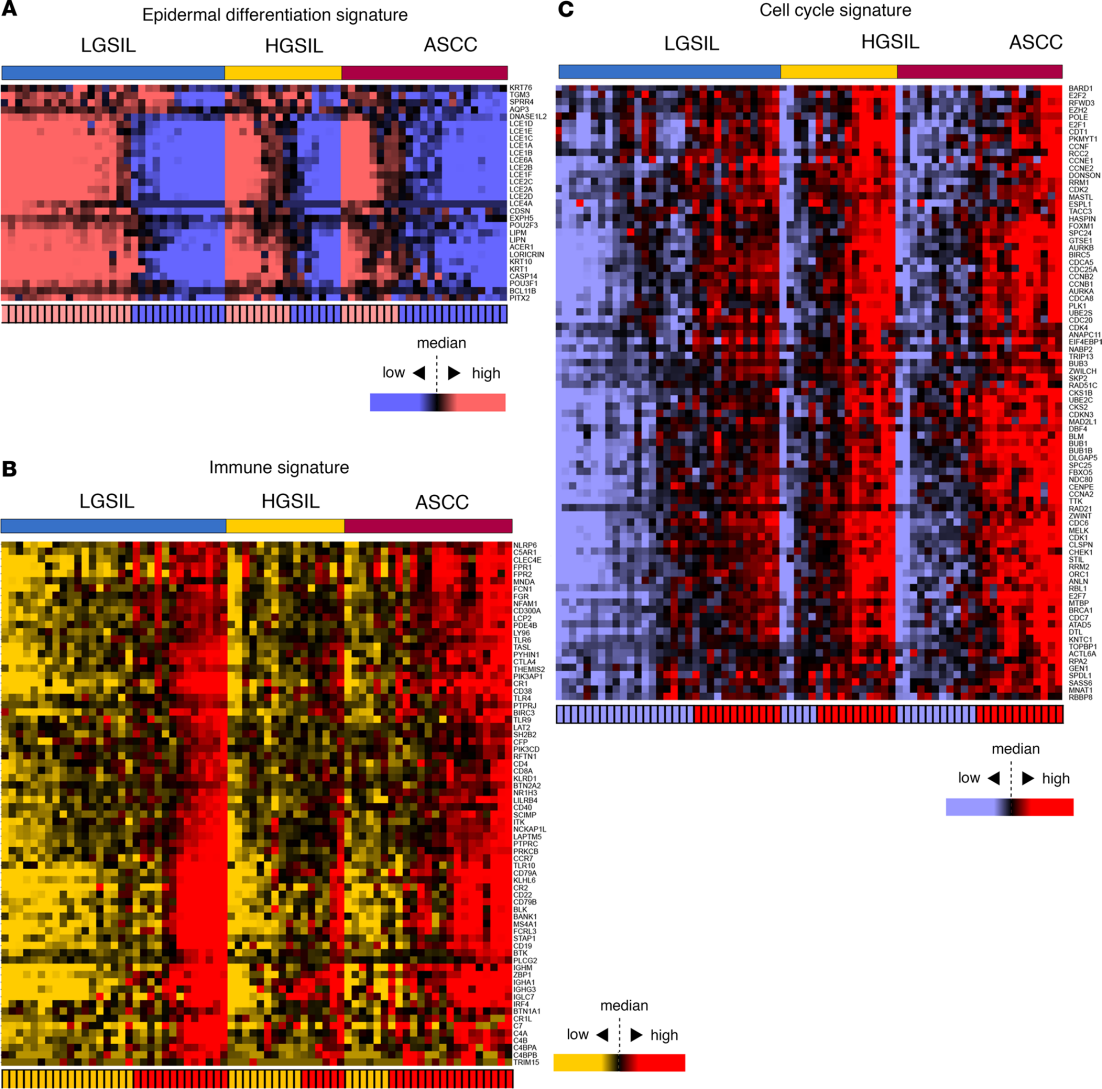
Heatmaps illustrating the expression profiles of gene signatures across diagnostic groups: LGSIL, HGSIL, and ASCC. (**A**) Epidermal differentiation signature. (**B**) Immune signature. (**C**) Cell cycle signature. The color coding bar at the bottom of each heatmap indicates the score (high or low) assigned to each sample based on the average expression of the gene signature divided by the median value.

GSEA highlighted deregulated processes across anal lesion stages, emphasizing central roles for the cell cycle, immune response, viral infection, and epidermal differentiation. We focused on significant gene signatures obtained by GSEA related to these processes to visualize gene expression patterns including epidermal differentiation (30 genes — Figure 5A and Supplemental Data 6), immune response (72 genes — Figure 5B and Supplemental Data 6), and cell cycle (86 genes — Figure 5C and Supplemental Data 6). Heatmaps revealed at least 2 subtypes within each diagnosis group, one with high expression of the gene signature and the other with low expression. To categorize samples, we introduced “high” and “low” scores based on the average expression of each gene signature, divided by the median value (Figure 5, A–C).

**Figure 6:**
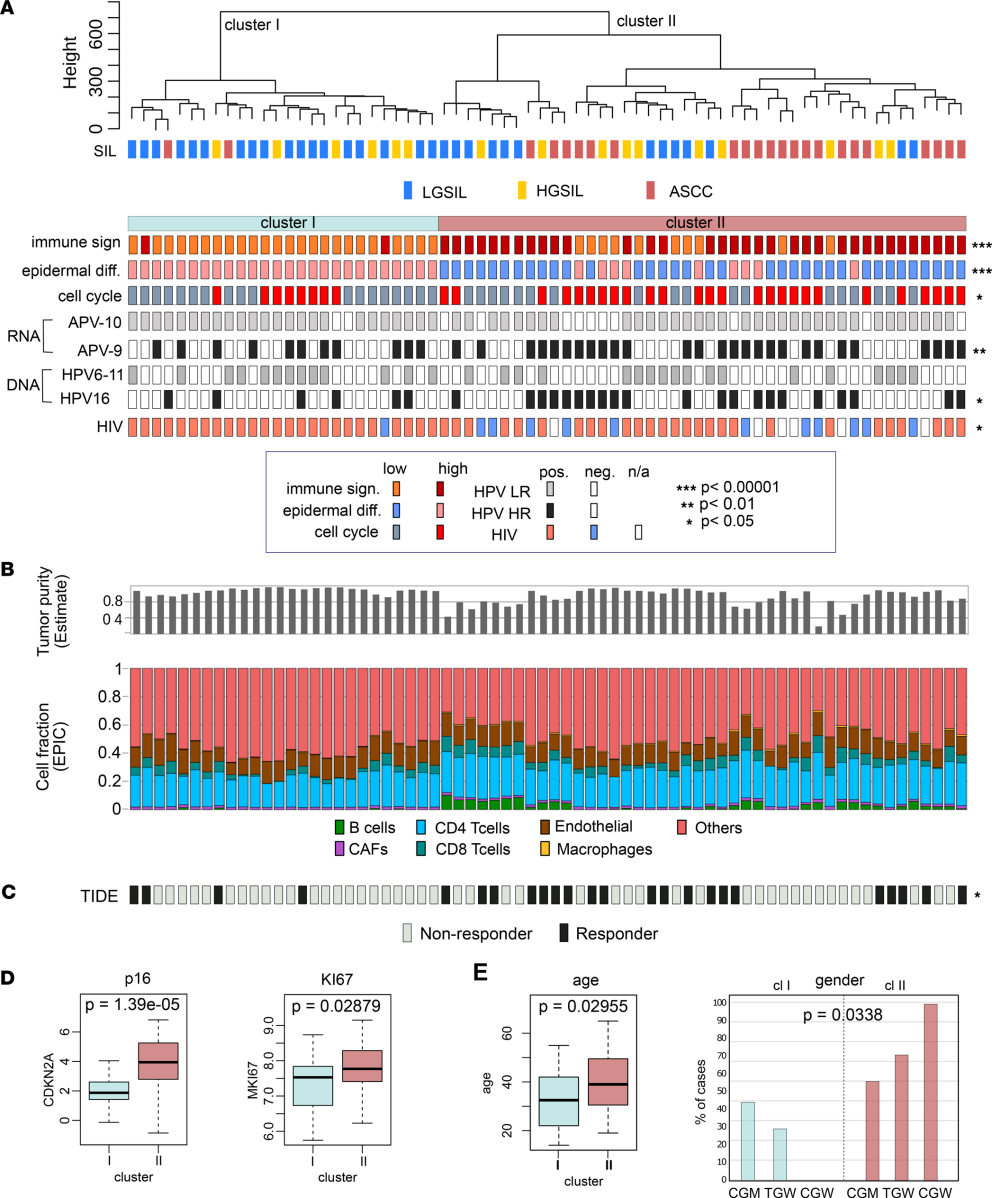
Integrative analysis of host transcriptome of LGSIL, HGSIL, and ASSC. (**A**) Tile plot illustrating signature scores, HPV status, and HIV status of samples distributed according to the unsupervised clustering analysis. Statistical significance was determined through the application of Fisher’s exact test. (**B**) Immune profiling and cell fraction composition for each sample using ESTIMATE and EPIC, respectively. (**C**) T cell dysfunction and exclusion (TIDE) score for each sample. Statistical significance was determined through the application of Fisher’s exact test. (**D**) Relative mRNA abundance of CDKN2A (p16) and MKI67 (Ki67) across samples in cluster I versus cluster II. (**E**) Comparative analysis of clusters for age and gender. Statistical significance was determined through the application of a *t* test for age and χ^2^ test for gender. *P < 0.05; **P < 0.01; ***P < 0.001.

Next, we incorporated these signatures along with LR- and HR-HPV and HIV status into the unsupervised clustering of samples. This allowed us to discern 2 primary clusters with distinct characteristics (Figure 6A). Cluster I primarily comprised SILs (*P* < 0.01; 24 out of 26 in cluster I) with a low immune signature (*P* < 0.001), high epidermal differentiation (*P* < 0.001), low cell cycle signature (*P* < 0.05), and smaller number of samples infected with HR-HPV types detected at both RNA (*P* < 0.05) and DNA (*P* < 0.05) levels compared with cluster II. In contrast, cluster II encompassed 91% of anal cancer cases (*P* < 0.01; 21 out of 23 ASCC) and 62% of HGSILs (10 out of 16 HGSILs). It exhibited a higher immune signature score (*P* < 0.001), low epidermal differentiation (*P* < 0.001), greater number of samples with a high cell cycle signature (*P* < 0.05), and higher prevalence of HR-HPV infections (*P* < 0.05; Figure 6A). Of note, cluster II included most of the participants without HIV (92%; 11 out of 12 HIV-negative cases) compared with cluster I (*P* < 0.05), which was mainly integrated with PLWH (25 out of 26 cases in cluster I).

### Immune infiltration and cell composition analysis.

We utilized EPIC and ESTIMATE algorithms for predicting immune infiltration and cell fraction composition (Figure 6B). Cluster II exhibited a higher level of immune infiltration, as determined by EPIC (*P* < 0.001). The analysis of cell composition revealed a significant increase in B cells (*P* < 0.001), CD4^+^ T cells (*P* < 0.001), CD8^+^ T cells (*P* < 0.05), and macrophages (*P* < 0.001), aligning with the high immune signature assigned to this cluster (Supplemental Data 7). A possible explanation for these findings could be the higher prevalence of HIV-negative cases in cluster II, suggesting a potentially less compromised immune system compared with individuals in cluster I.

To explore further, we conducted a comparison of the immune profile between HIV-positive and HIV-negative individuals, irrespective of their cluster assignment. Results revealed a significant reduction in B cells (*P* < 0.01) and CD4^+^ T cells (*P* < 0.001) among PLWH in our cohort (Supplemental Data 7). This aligns with the asymptomatic phase of HIV infection, characterized by ongoing viral replication leading to a gradual depletion of CD4^+^ T cells, which can be partially restored with ART. While the impact of HIV on B cell numbers is less clear, studies indicate a reduction in B cell counts in HIV-infected individuals (39). Dysregulation of B cells during HIV infection is also influenced by ART. Of note, a substantial portion of individuals in our HIV-infected cohort were on ART during recruitment, contributing to observed variations in B cell composition.

Furthermore, we explored whether there was an association between these immune profiling differences and HPV16 infection. Results indicated a significantly higher immune profile of macrophages in HPV16-infected cases (*P* < 0.01; Supplemental Data 7). Previous studies have reported that M2-like macrophages infiltrate HPV16-associated tumors, suppressing antitumor T cell response and facilitating tumor growth (40).

Overall, cluster II was represented by ASCC tumors and precancerous lesions with a high immune infiltration. The significance of tumor-infiltrating lymphocytes (TILs) in influencing favorable outcomes across various tumor types, including ASCC, has been reported in the literature (41–44). Our recent study demonstrated the crucial role of PD-L1 expression in influencing complete response rates and survival outcomes in patients with nonmetastatic ASCC undergoing standard definitive chemoradiotherapy (17). Motivated by the importance of immune factors in ASCC, we used the T cell dysfunction and exclusion score (TIDE) in our study to predict cancer immunotherapy response.

The results yielded a compelling connection between immune-related characteristics and treatment response. Cluster II, characterized by a higher immune signature and immune cell infiltration, exhibited a significantly higher number of responders (*P* < 0.05; Figure 6C). The TIDE analysis highlighted specific immune cell changes associated with responders, including an increase in CD4^+^ TILs (*P* < 0.05) and macrophages (*P* < 0.05), and a concurrent decrease in cancer-associated fibroblasts (CAFs, *P* < 0.01) and endothelial cells (*P* < 0.01) (Supplemental Data 7). These findings underscore the potential predictive value of immune-related parameters in discerning responders and nonresponders to cancer immunotherapy in the context of anal cancer progression.

Furthermore, we compared the gene expression profiles of 2 surrogate markers for HPV-related malignancy, Ki67 and p16. Results showed that both markers were higher in cluster II (Figure 6D). Additionally, cluster I was linked to younger participants and MSM, while cluster II was associated with older patients and enriched in TGW and CGW (Figure 6E). Coinciding with the latter, high p16 expression has been shown to correlate with female sex and better outcomes following chemoradiotherapy (45–47).

These findings might help to better understand the molecular landscape within and between different stages of anal lesions and reveal potential biomarkers and therapeutic pathways for further research.

### Immune profiling of p16^+^ and CD3^+^/CD8^+^ cells and PD-L1 expression among ASCC.

The immunohistochemical (IHC) analysis of p16, CD3, CD8, and PD-L1 in anal cancer not only provides valuable insights into the tumor microenvironment but also serves to guide treatment decisions and prediction of patient outcomes (17).

**Figure 7:**
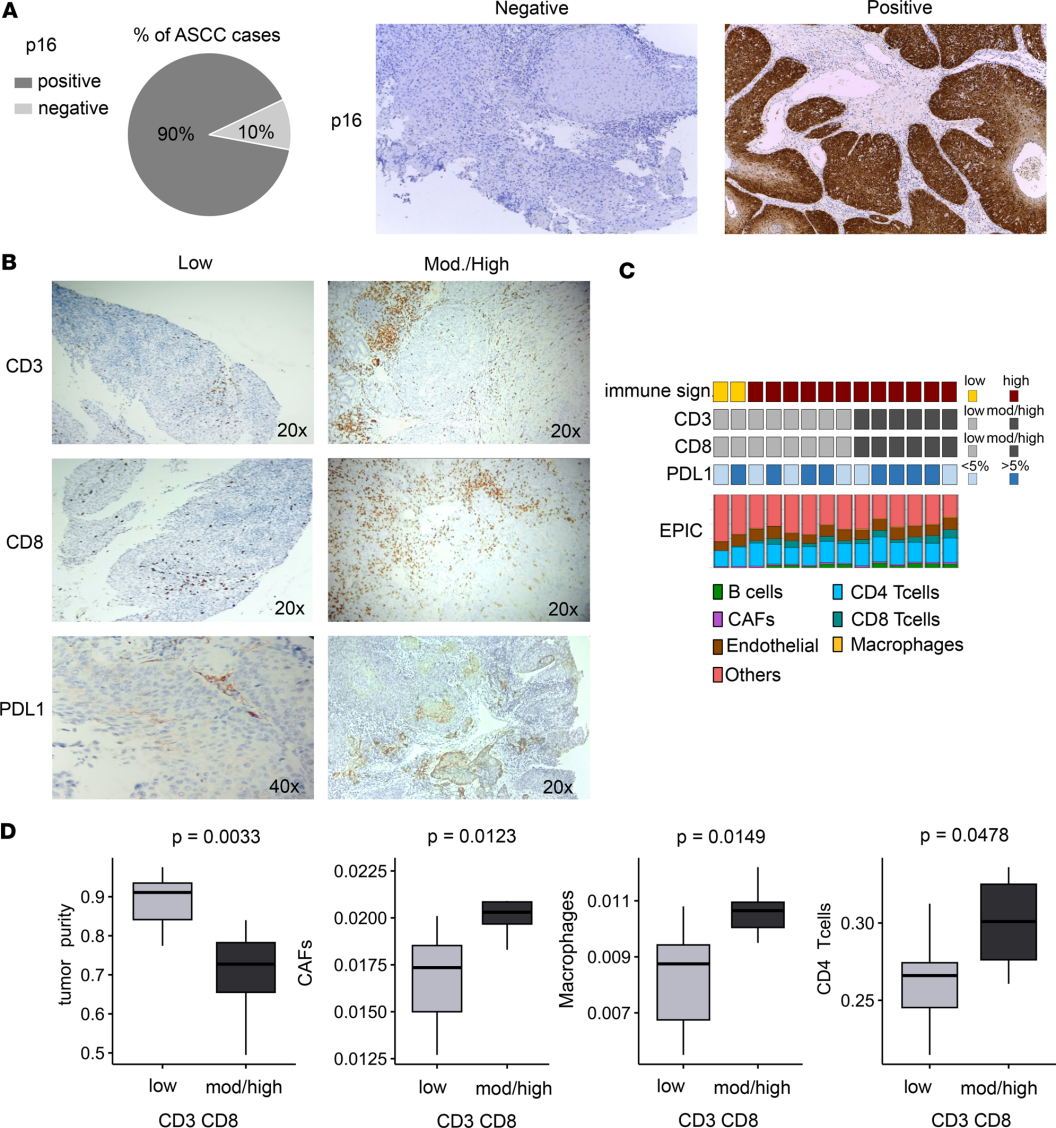
Comprehensive analysis of p16^+^, CD3^+^, and CD8^+^ TILs’ density and PD-L1 expression in the tumor microenvironment of ASCC. (**A**) Immunohistochemistry (IHC) results of p16 in 10 ASCC cases. Microphotographs represent negative and diffusely positive p16 staining on ASCC (original magnification, ×10). (**B**) Representative IHC results depicting high and low expression levels of CD3, CD8, and PD-L1. (**C**) Tile plot illustrating ASCC samples analyzed by IHC, showcasing scores for immune signature; CD3, CD8, and PD-L1 IHC results; along with EPIC cell fractions. (**D**) Box plots comparing tumor purity, CAFs, and macrophage levels, as obtained by EPIC, between tumors with low (*n* = 8) and high (*n* = 6) CD3^+^/CD8^+^ TILs. Statistical significance was calculated with Wilcoxon’s signed-rank test.

In our study, we explored these markers in 10 (for p16) and 14 (for CD3, CD8, and PD-L1) out of the 23 ASCC samples using IHC. Ninety percent of ASCC (9 out of 10) showed a diffusely positive pattern of p16 (Figure 7A). The density of CD3^+^ and CD8^+^ TILs was moderate to high in 47% (6 out of 14) of ASCC samples (Figure 7B). Of note, all these samples exhibited a high immune signature, correlating with increased immune infiltration as assessed by EPIC (Figure 7C). In this context, tumors with moderate to high CD3 and CD8 expression were associated with lower tumor purity scores (*P* < 0.01) and higher cell fractions of CAFs (*P* < 0.05), macrophages (*P* < 0.05), and CD4^+^ T cells (*P* < 0.05) as revealed by EPIC analysis (Figure 7D). The PD-L1 expression status was assessed in the 14 ASCC cases using the Combined Positive Score (CPS). Notably, 57% of positive cases (8 out of 14) exhibited moderate to high PD-L1 expression levels (CPS > 5%), while the remaining samples showed low PD-L1 expression levels (CPS < 5%; 6 out of 14) (Figure 7B). This analysis indicates a complex relationship between TILs and tumor microenvironment factors, shaping the immune profile of ASCC tumors and potentially influencing treatment approaches.

### Comparative transcriptome analysis of HPV-related squamous cell carcinomas.

**Figure 8:**
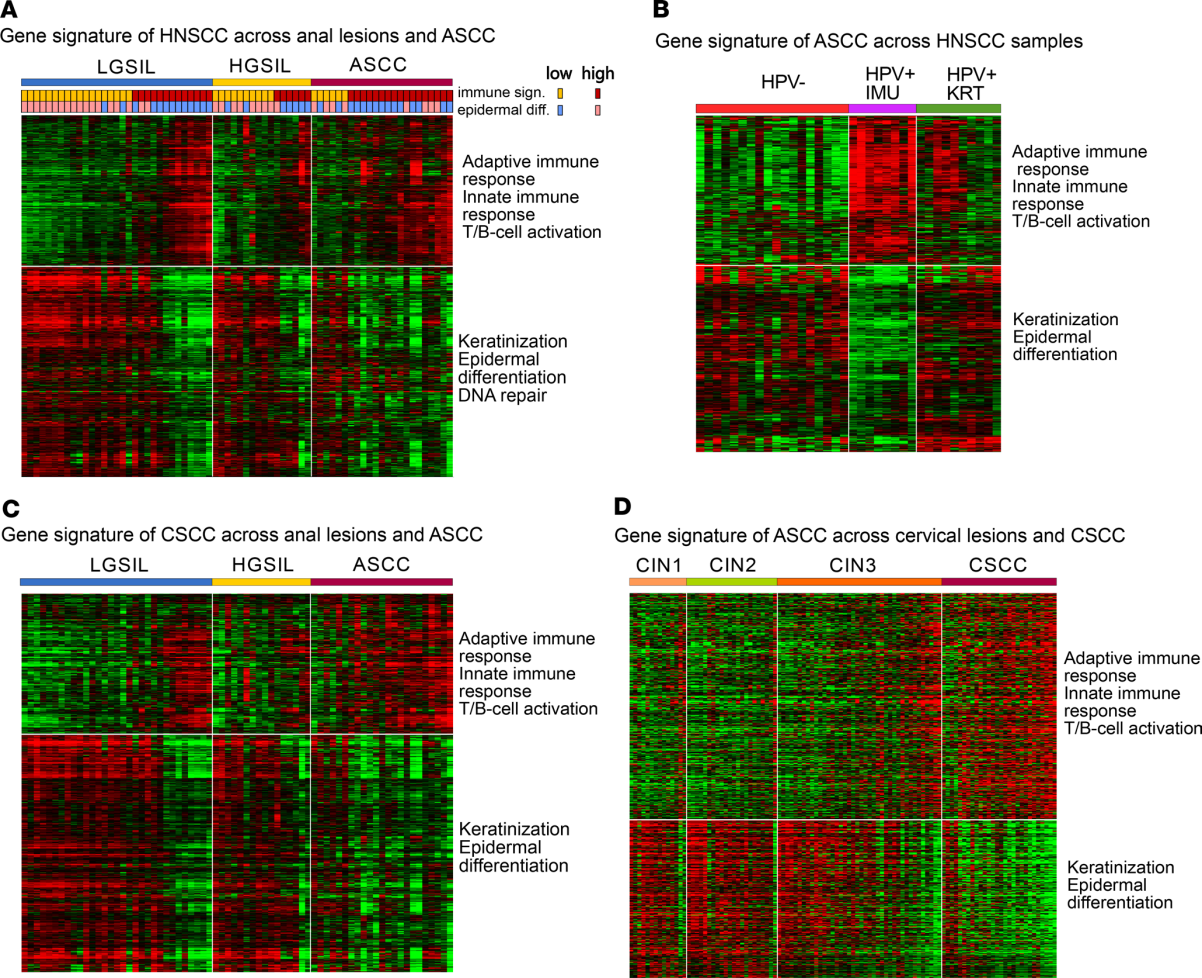
Comparative analysis of gene signature expression patterns and enriched pathways in HNSCC, cervical lesions, and anal lesions. (**A**) Heatmap visualization of HNSCC gene signature across our sample cohort, grouped by immune score within each diagnosis category. Additionally, the epidermal differentiation score is displayed. (**B**) Heatmap visualization of the ASCC gene signature expression profile in HNSCC samples organized by subtype classification according to Zhang et al., 2016 (48). (**C**) Heatmap visualization of CSCC gene signature across our sample cohort grouped by immune score within each diagnosis category. (**D**) Heatmap visualization of the ASCC gene signature across cervical lesions, arranged in ascending order based on the immune gene profile within each diagnosis category.

We analyzed relevant HPV-associated cancer studies to compare the gene expression signatures identified in ASCC with head and neck squamous cell carcinomas (HNSCCs) and cervical squamous cell carcinomas (CSCCs). In a previous study, Zhang et al. conducted RNA-Seq on 36 HNSCCs (18 HPV^+^ and 18 HPV^–^), identifying 2 HPV^+^ subtypes. One subtype was enriched in “immune response” genes; the other was enriched in “keratinocyte differentiation” genes (48), consistent with our ASCC findings. We applied the gene signature distinguishing these subtypes in HNSCC across our sample cohort, sorted by immune score (Figure 8A and Supplemental Data 8). Additionally, we employed our gene signature, derived from the most significantly deregulated genes in the HGSIL versus ASCC comparison, on HNSCC samples, grouped by the subtypes defined by the authors (Figure 8B and Supplemental Data 8). Results indicated similar gene expression patterns between locations, with variations in gene composition, yet aligned with similar biological processes. For CSCC, we utilized den Boon et al.’s study, despite being microarray based, because of its comprehensive analysis of premalignant (CIN1, CIN2, and CIN3) and CSCC specimens (49). We established a gene signature by comparing CIN2/CIN3 (comparable to HGSIL) versus CSCC and visualized the gene expression profile in our sample cohort (Figure 8C and Supplemental Data 8). This analysis and the application of our signature to cervical lesion samples, sorted by immune score (Figure 8D and Supplemental Data 8), showed an almost mutually exclusive relationship between immune and epidermal differentiation processes. This suggests a significant decrease in keratinocyte differentiation as the disease progresses, alongside a significant increase in immune response genes.

### Mutational profiling of cancer driver genes among ASCC and other squamous cell carcinomas.

**Figure 9:**
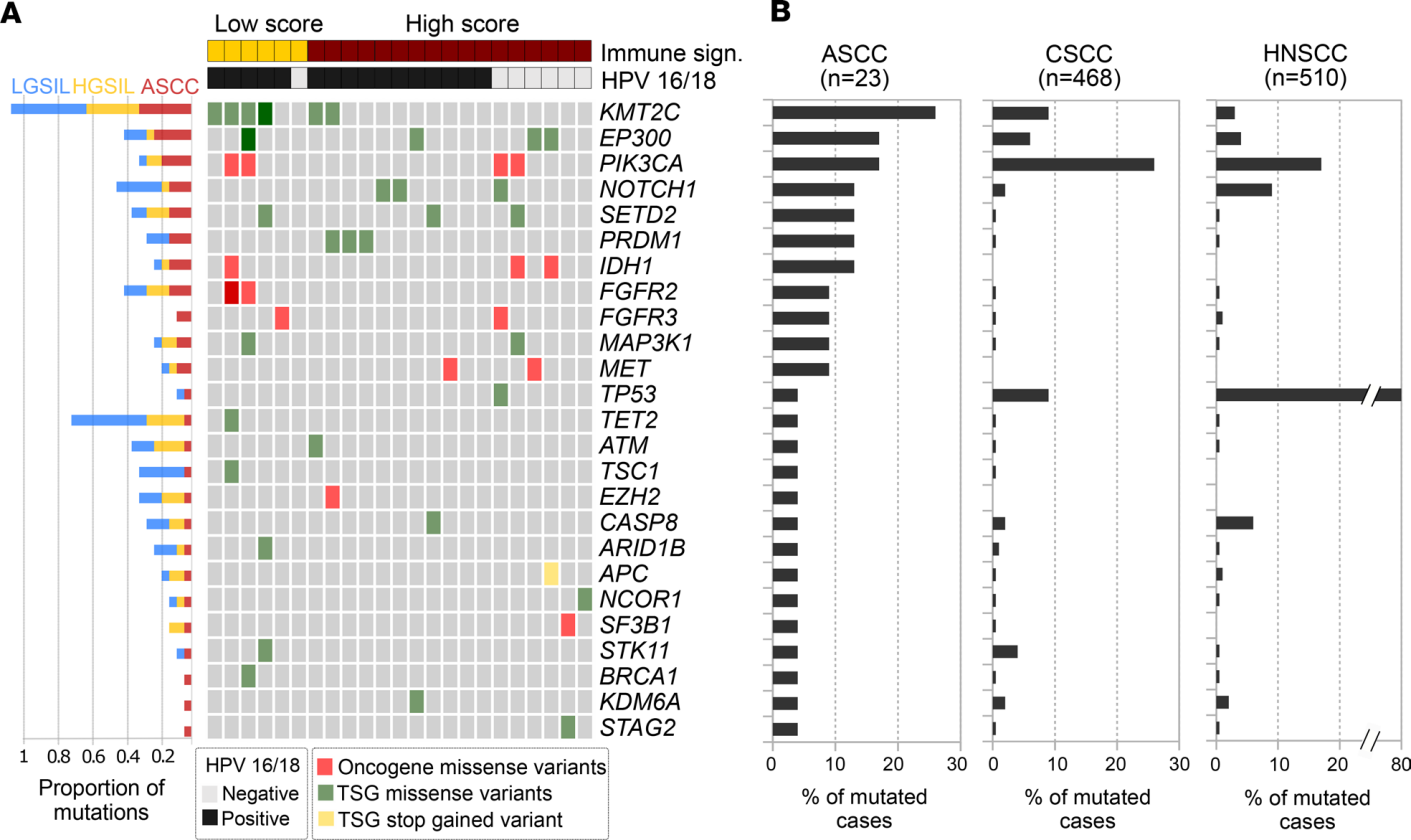
Mutational profiles among squamous cell carcinomas. (**A**) Tile plot of the most prevalent somatic cancer driver mutations identified in 23 ASCC cases through transcriptome-based sequencing. The upper color-coded bars provide an indication of the immune signature score and HR-HPV status for each respective sample. On the left bar plot, the proportions of somatic mutations within each group are presented, relative to the total number of cases in that specific group. TSG, tumor suppressor gene. (**B**) Comparative frequency of the mutations identified in the ASCC cohort with respect to CSCC and HNSCC retrieved from the TCGA cohorts.

We conducted mutational profiling on ASCC biopsies from 23 patients based on RNA-Seq data, revealing 51 somatic missense mutations in cancer driver genes among 87% of ASCC cases (20 out of 23). We identified mutations in lysine methyltransferase 2C (*KMT2C*, also known as *MLL3*, 30%), phosphatidylinositol-4,5-bisphosphate 3-kinase catalytic subunit alpha (*PIK3CA*, 20%), *EP300* (chromatin remodeler, 20%), *NOTCH1* (15%), *IDH1* (15%), PR/SET domain 1 (*PRDM1*, 15%), *FGFR2* (15%), *SETD2* (15%), *FGFR3* (10%), *MAP3K1* (10%), and *MET* (10%). Single cases of mutations were found affecting *TP53*, *TET2*, *ATM*, *TSC1*, *EZH2*, *CASP8*, *ARID1B*, *APC*, *NCOR1*, *SF3B1*, *STK11*, *BRCA1*, *KDM6A*, and *STAG2* (Figure 9A). Several of these mutated genes are commonly found in HPV-driven squamous cancers like cervix, head and neck, vulva, and anus, including *KMT2C*, *EP300*, *PIK3CA*, *NOTCH1*, *FGFR2*, *ATM*, *TP53*, and *BRCA1* (17, 50, 51).

Consistent with our results, comparable frequencies of *KMT2C*, *PIK3CA*, and *EP300* have been reported at the genomic level through NGS or targeted sequencing among the most mutated genes in ASCC (17, 51–53).

Our data revealed *KMT2C* mutations at comparable rates in the early stages of anal lesions, reaching 30% in HGSIL and 42% in LGSIL (Figure 9A), suggesting a potential pivotal role for *KMT2C* as a driver gene in anal carcinogenesis progression. Additionally, increased mutation frequencies for *EP300* (21% in ASCC, 4% in HGSIL, and 13% in LGSIL) and *PI3KCA* (17% in ASCC, 8% in HGSIL, 4% in LGSIL) were observed compared with earlier stages of anal lesions (Figure 9A), indicating potential shifts in the molecular landscape during disease progression.

A higher mutation rate of 3.5 (21 mutations in 6 samples) was observed in ASCC with a low immune signature compared with the high–immune signature group (*P* < 0.01), which had a mutation rate of 1.76 (30 mutations in 17 samples). This implies distinct tumor subpopulations with mutations in cancer driver genes (Figure 9A).

Furthermore, all mutations in *KMT2C* (7 mutations in 6 cases), *PRDM1* (3 mutations in 3 cases), and *FGFR2* (3 mutations in 2 cases) occurred in HPV16-infected cases, comprising 25% of total mutations (Figure 9A). *PRDM1* is a master regulator of lymphoid cell differentiation and a tumor suppressor gene in lymphoma (54). It has been identified as a master regulator for HPV16 E6/E7 proteins (55) Aberrant *FGFR* signaling and HPV16 E5 expression have been shown to be correlated with cervical cancer progression (56). Furthermore, the interaction between HPV16 E5 and *FGFR2* alters keratinocyte differentiation and inhibits tumor-suppressive genes, suggesting a role in the early stages of HPV infection and transformation (56).

Consistent with our findings, previous studies have recognized *KMT2C* and *EP300* as the most frequently mutated genes in metastatic ASCC (51). *KMT2C* mutations are associated with abnormal H3K4 methylation, linked to oncogenic transformation in preclinical models (57). *KMT2C* plays a crucial role in activating *TP53* gene expression, demonstrated by targeted inactivation studies in mice (58).

Regarding *EP300*, the oncoprotein HPV E6 mediates *TP53* degradation by binding to the histone acetyltransferase *EP300*, inhibiting *EP300*-mediated *TP53* acetylation and promoting *TP53* degradation (59, 60). Consequently, dysregulated histone/chromatin modulation within the context of impaired DNA repair mechanisms emerges as a driver of malignancy. We categorized mutated genes into Cancer Hallmarks and observed that genome instability predominated (Supplemental Data 9). Genes like *KMT2C*, *EP300*, *IDH1*, *SETD2*, *TET2*, *BRCA1*, *TP53*, *APC*, *ATM*, *KDM6A*, *NCOR1*, *SF3B1*, and *STAG2* defined a gene network critical for ASCC, regardless of HPV infection, aligning with *TP53* association with HPV-HR negativity in our study, consistent with prior research (17, 51, 52).

To perform a comparative analysis of the mutational profile identified in ASCC with other squamous cell carcinomas, we analyzed 2 combined cervical cancer data sets (MSK-CESC and TCGA-CESC) and a head and neck cancer data set (TCGA-HNSC) retrieved from cBioPortal (http://www.cbioportal.org/). Only drivers and putative drivers’ somatic missense or truncating mutations were considered for frequency estimations among cohorts. The comparative analysis showed that one-third of the most frequent cancer driver mutations identified in ASCC (8 out of 25 genes) were also frequently mutated (>5% of cases) in CSCC and HNSCC (*KMT2C*, *EP300*, *PIK3CA*, *NOTCH1*, *TP53*, *CASP8*, *STK11*, and *KDM6A*) (Figure 9B).

Our mutational profiling of ASCC biopsies from 23 patients offered valuable insights into the somatic mutation landscape of cancer driver genes, particularly given their derivation from transcriptomic data. However, we recognize the significance of the limited sample size when drawing definitive conclusions.

## Discussion

ASCC represents only 2% of all gastrointestinal tumors but is characterized by high morbidity and mortality. Unfortunately, treatment options for ASCC have not evolved in the past 20 years; concurrent chemoradiotherapy continues to be the standard care strategy for nonmetastatic cases. For patients with metastasis at diagnosis or those who develop metastatic recurrences after chemoradiation therapy, the 5-year survival rate is below 20% (61). To date, platinum-based chemotherapy doublets are the most commonly used anticancer drugs for palliative chemotherapy, and no targeted agents have been approved. In clinical practice, prognostic factors of survival in ASCC are the T and N stage, sex, differentiation, tumor location, HR-HPV infection, and occurrence of a complete response after chemoradiation therapy (17). These clinical parameters related to survival cannot be used to personalize therapy or predict treatment response in individual patients. Less is known regarding early-stage prognostic biomarkers of ASCC.

Comprehensive characterization of anal squamous precancerous and cancerous lesions at metatranscriptome and transcriptome levels allowed us to identify the most relevant changes in the cell host and their associated microenvironment — the immune infiltrate and the microbiome — during progression from preinvasive to invasive stages. Unsupervised analyses allowed us to identify 2 patient clusters (cluster I and cluster II) based on histological diagnosis, microbial composition, cell cycle, immune infiltrate, immune response, viral infection (HIV and HPV), epidermal differentiation, and activity of specific metabolic and signaling pathways. Cluster I was mainly composed of LGSIL and HGSIL differentiated and lowly proliferative cases with low immune infiltrate and almost all infected by LR-HPV types. Meanwhile cluster II was significantly enriched in ASCC and HGSIL cases with higher immune signature score, low epidermal differentiation, a greater number of samples with a high cell cycle signature, and a higher prevalence of HR-HPV. In this sense, cluster II was associated with higher expression of Ki67 and p16, older patients, TGW, and CGW. These findings align with previous studies that have implicated specific viral infections, immune responses, and molecular pathways in the progression of anal lesions (4, 10, 17). The observed distinctions between cluster I and cluster II provide valuable insights into potential prognostic and therapeutic considerations in the management of anal squamous lesions (62).

### Microbiome changes in preinvasive and invasive stages of anal cancer.

A comparison of the microbiota composition at phylum and species levels revealed expected differences between SILs and ASCC regarding the prevalence of HR-HPV subtypes but also identified several viruses and bacteria species significantly associated with anal cancer not previously reported to our knowledge. In this sense, *F*. *nucleatum*, *F*. *gonidiaformans*, and *B*. *fragilis,* previously associated with CRC progression at early stages (17), were significantly enriched in ASCC compared with premalignant lesions. More importantly, these taxa together with HPV16 contributed to gene-encoding enzymes (e.g., Acca, glyQ, eno, pgk, and por) and oncoproteins (FadA and dnaK) and a distinctive ASCC metabolic profile characterized by the enrichment of pathways related to oxidative, energetics, or biosynthetic processes, including glycolysis, lipid, amino acid, and nucleotide biosynthesis, that could facilitate and promote the survival and proliferation of cancerous cells (10, 29, 30). Among these enzymes and proteins, Acca, glyA, glyQ, eno, pgk, and por were identified in our previous study as associated with precancerous anal lesions, highlighting their roles as metabolic markers in cancer progression (10). In line with our results, Serrano and Villar also found pgk and eno overexpressed in the microbiome of HGSIL, while they proposed succinyl-CoA and cobalamin as markers associated with HGSIL (8). This reinforces the idea that HPV-infected cells can modify metabolism by regulating genes involved in cellular growth and metabolism, which is crucial to oncogenesis (63). Considering and validating these microbial proteins as markers could offer alternative tools in cancer prevention.

### Cell signaling pathways affected in preinvasive and invasive stages of anal cancer.

Our integrative analysis of the host transcriptome provided valuable insights into the molecular landscape underlying anal cancer development. The transition from HGSIL to ASCC was characterized by a statistically significant number of DEGs, with notable alterations in keratin expression and overexpression of members of the MAGE gene family in ASCC. Functional analysis revealed key biological processes and pathways associated with each stage. In HGSIL compared with LGSIL, activated processes included nuclear division, chromatin modification, and cell proliferation, aligning with histopathological features indicative of high-grade lesions (35). Conversely, the transition from HGSIL to ASCC revealed immune response activation, marked by upregulation of IFN pathways, highlighting the role of the immune system in the progression to anal squamous cell carcinoma (38). Indeed, patients of cluster II were characterized by a higher immune signature and immune cell infiltration, as assessed by gene expression profiling of immune cell fractions, IHC of CD3^+^ and CD8^+^ TILs, as well as PD-L1 expression.

Therefore, in comparing HGSIL and ASCC, the data underscored the predominance of immune response activation in ASCC, contrasting with the cell proliferation and DNA modification processes observed in the transition from LGSIL to HGSIL. Noteworthy findings included the suppression of the p53 pathway potentially linked to the overexpression of HPV16 E6 protein, highlighting the intricate interplay between viral oncoproteins and host cellular processes in the progression to ASCC (64).

### Shared and unique immune and molecular changes across squamous cell carcinomas.

Through the integration of transcriptomic studies on HNSCC and CSCC with our ASCC transcriptome, we found shared gene expression patterns across tumor sites indicating a shift toward immune response genes and a decrease in keratinocyte differentiation genes during disease progression from preinvasive to invasive stages. These patterns align with the known biology of HPV carcinogenesis, where HPV E6 oncoprotein downregulates keratinocyte differentiation genes and upregulates mesenchymal lineage genes (65). Regarding the immune response, the elevated immune score and the high frequency of TIL cell fraction observed in cluster II samples from our analysis are also in line with a higher prevalence of HR-HPV. HPV-positive tumors may have increased numbers of TILs, myeloid dendritic cells, and proinflammatory chemokines, which are thought to improve treatment response in patients with head and neck and cervical cancers (66, 67). Our results showed that a strong immune response is associated with better treatment outcomes, as indicated by TIDE score analysis. Studies have shown that TILs may improve treatment responses or outcomes in patients with CSCC undergoing chemotherapy or radiotherapy (68, 69). TIL-based immunotherapy has shown promise as an alternative treatment for advanced cervical cancer, with positive results (70). In advanced ASCC, immunotherapy trials primarily focus on targeting PD-1/PD-L1 and E6/E7 proteins (71). Therefore, combining TIL therapy with checkpoint blockade and HPV E6/E7 vaccination offers a potent antitumor therapy with the potential to eradicate malignancy in ASCC completely.

Furthermore, ASCC exhibited somatic missense mutations in cancer driver genes, with *KMT2C*, *PIK3CA*, and *EP300* being the most mutated genes, in agreement with previous reports. The prevalence of these mutations varied at different stages of anal precancerous lesions, suggesting their involvement in early stages of anal cancer development. Additionally, there were distinct tumor subpopulations with different mutation rates and immune signatures. Mutations in *KMT2C*, *PRDM1*, and *FGFR2* were predominantly found in HPV16-infected cases, indicating their association with HPV-related carcinogenesis (51, 52). The comparative analysis of mutational profiles across different squamous cell carcinomas, including ASCC, HNSCC, and CSCC, revealed substantial overlaps in the mutation landscape. By examining data sets from cBioPortal, encompassing a substantial number of cases, we identified common mutations in several cancer driver genes among these carcinomas. Approximately one-third of the frequently mutated genes in ASCC were also prevalent in HNSCC and CSCC, suggesting potential shared molecular mechanisms underlying these cancers. Key drivers such as *KMT2C*, *EP300*, *PIK3CA*, *NOTCH1*, *TP53*, *CASP8*, *STK11*, and *KDM6A* emerged as recurrently altered across these cohorts. However, the remaining two-thirds of the mutated genes appeared to be specific to ASCC, indicating distinct genetic alterations driving the development and progression of anal cancer.

Our study has a number of limitations given its cross-sectional nature and the low sample size utilized for data collection because of the rarity of anal cancer. The small sample size might not fully represent the biological diversity and variability within the population under investigation, potentially limiting the generalization of the findings. Furthermore, the high risk of false discovery poses a considerable concern, especially in exploratory analyses or when multiple comparisons are conducted. Due to the cross-sectional design adopted in this study, establishing causal associations becomes challenging. However, we believe this is the first cross-sectional study that identifies metatranscriptomic and transcriptomic changes among premalignant and malignant stages of anal cancer. Furthermore, these findings provide valuable insights into novel prognostic biomarkers that may help stratify patients with precancerous lesions in low- versus high-risk groups of progression to the malignant stage. Future research employing larger sample sizes and longitudinal designs would be needed to address these limitations and corroborate our findings.

## Methods

### Sex as a biological variable.

Sex at birth (male or female) and gender identity (CGM, TGW, and CGW) were incorporated into our study design as biological variables.

### Sample collection and RNA-Seq.

We collected 70 anal biopsies from patients with different stages of anal lesions: 31 LGSIL, 16 HGSIL, and 23 ASCC, stored in RNAlater (Thermo Fisher Scientific). Clinical data including age, HPV status, ART treatment, and HIV status were recorded at enrollment. RNA was extracted using miRNeasy Tissue/Cells Advanced Kits (QIAGEN), and its quality was assessed on an Agilent 2100 Bioanalyzer. Samples with RNA integrity number greater than 7 were chosen for RNA-Seq. Directional RNA-Seq libraries were prepared using Illumina Total RNA Prep with Ribo-Zero Plus kit. Sequencing was performed on an Illumina NovaSeq 6000 platform, yielding approximately 80 million clusters per sample with >92% >Q30 quality scores.

### DNA purification and HPV detection and genotyping.

Samples were collected using QIAGEN specimen collection devices by qualified staff at Fundación Huésped and Hospital Udaondo. DNA purification utilized QIAamp DNA Kits (QIAGEN). DNA integrity and concentration were assessed by NanoDrop spectrophotometry (Thermo Fisher Scientific). HPV detection was performed at Institute Malbrán via PCR using biotinylated Broad-Spectrum General Primers BSGP5^+^/GP6, designed to amplify a 140 bp fragment of the HPV-L1 gene. Genotyping was conducted using reverse line blot hybridization for 36 HPV genotypes (validated by Global HPV LabNet) (72). Biotinylated amplicons were denatured and hybridized with genotype-specific oligonucleotide probes immobilized as parallel lines on membrane strips.

### Metatranscriptomic data analysis.

For metatranscriptomics, the obtained RNA-Seq data were processed using the bioBakery suite of tools: KneadData was used to separate the human and the nonhuman reads; taxonomic profiling was performed using MetaPhlAn to identify and quantify microbial taxa at species level present in the anal samples (73).

Species richness and diversity were calculated using the R function estimate_richness from R package phyloseq (74). We considered the observed species and Chao1 indices for richness and the Shannon and Simpson indices for diversity. We measured β-diversity by Bray-Curtis, weighted UniFrac, and unweighted UniFrac. For PCoA, the Aitchison distance was used as the distance metric to analyze the compositional data. To test whether the samples cluster beyond that expected by sampling variability, we applied PERMANOVA. Differences in richness and diversity indices between groups were determined using the Wilcoxon rank-sum test with a significance level of 0.05. For relative abundance analysis and visualization, we used R phyloseq packages.

### Differential abundance analysis.

For determining the relative differential abundance and the multivariable association between participants’ metadata and microbial features, we used the MaAsLin2 package from the bioBakery suite in R/Bioconductor (75). We used default parameters for normalization (total sum scaling method), transformation (log), analysis method (linear models), correction method (Benjamini-Hochberg), and significance threshold (*q* value < 0.25). The minimum abundance for each feature was set to 0.001 (0.1%) while the minimum percentage of samples for which a feature was detected (prevalence) at minimum abundance was used as follows: 0.05 (5%) for viruses, 0.1 (10%) for bacteria and pathways, and 0.2 (20%) for gene families.

### Pathways and gene family analysis.

Metatranscriptomic pathway analysis was conducted using the HMP Unified Metabolic Analysis Network 3 (HUMAnN3) pipeline to investigate potential variations in metabolic pathways. HUMAnN3 employs a multifaceted approach, extracting species profiles from KneadData output, aligning reads to pan-genomes, executing translated searches on unclassified reads, and quantifying gene families and pathways. By default, gene families are UniRef90 annotated and metabolic pathways are annotated using MetaCyc database (76, 77). The UniRef90 gene family abundance from HUMAnN3 was then regrouped to KEGG orthology (KO) (78). We used the KEGGREST package in R/Bioconductor for KO identifiers and KEGG Mapper reconstruct tool for KEGG pathway maps (79, 80).

### Data visualization.

We used the R package phyloseq to create a heatmap representation of taxa abundances. For the unsupervised ordination of samples, we applied the nonmetric multidimensional scaling method and Bray distance in the plot_heatmap function. Heatmap visualization of differentially represented gene proteins was done with R/Bioconductor and the MultiExperiment Viewer software (MeV v4.9).

### Transcriptomic, functional enrichment, and immune infiltrate analysis.

The raw short-read sequences were preprocessed using Rfastp from the R/Bioconductor package Rsubread. Quality checks, adapter removal, and trimming of low-quality bases were conducted with Rfastp. Reads were aligned to the human genome reference GRCh38 using the Subread aligner algorithm from Rsubread. Gene expression abundance at the whole-genome level was calculated using featureCounts from Rsubread. Differential gene expression analysis between anal lesion stages (LGSIL vs. HGSIL and HGSIL vs. ASCC) utilized edgeR, with FCs and adjusted *P* values computed based on normalized log_2_ count per million values. Genes with a log-FC > 1 and adjusted *P* < 0.05 were considered differentially expressed.

Functional enrichment analysis of DEGs employed the clusterProfiler package for GSEA (81). Functional enrichment results were visualized using enrichplot for GO, Hallmark of Cancers, and DO terms. Heatmaps were generated using MeV 4.9.0.

Tumor purity, immune cell infiltration, and T cell dysfunction/exclusion scores were estimated using ESTIMATE, EPIC, and TIDE algorithms, respectively, on normalized count matrices.

For comparative transcriptomics analysis, the NCBI Gene Expression Omnibus GSE74927 data set for HNSCC and GSE63514 for CSCC were utilized. Raw data were imported into R using GEOquery to obtain normalized matrices for each study. Differential gene expression analysis employed DESeq2 for GSE74927 and limma for GSE63514. To visualize gene expression patterns, we defined the following gene signatures: for ASCC, we used the gene signature obtained from our comparison of HGSIL versus ASCC; for HNSCC, we used the gene signature provided in the study by Zhang et al., derived from the differential expression analysis between 2 HPV^+^ subgroups of HNSCC (48); for CSCC, we obtained a gene signature from the comparison between CIN2/CIN3 samples (comparable to HGSIL) and CSCC. Gene expression profiles across ASCC, HNSCC, and CSCC matrices were visualized after filtering out genes with less than 50% variance within each signature. Functional enrichment analysis of resulting genes used the clusterProfiler package. Heatmaps were generated in MeV 4.9.0 based on immune scores.

### Mutational analysis based on RNA-Seq data.

The preprocessed reads previously used for the transcriptomic analysis were aligned and mapped to the human genome reference GRCh38 using the Subjunc aligner algorithm provided by Rsubread R/Bioconductor package. Subjunc aligner was developed for aligning RNA-Seq reads and for the detection of exon-exon junctions at the same time. The Subjunc mapping results (BAM files) were used for genomic variant detection using the exactSNP variant caller algorithm provided by Rsubread package. The VariantAnnotation R/Bioconductor package was subsequently used for SNP and indel filtering of the obtained VCF files based on quality (–log_10_[*P* value] > 20) and coverage (read depth > 10) metrics. Identified variants were annotated, filtered, and interpreted using OpenCRAVAT and their aggregated variant databases and resources (gnomAD, Cancer Genome Interpreter, Cancer Hotspots, CIVIC, Cosmic, SIFT, PolyPhen2) for the prediction of somatic mutations in cancer driver genes.

In addition, to perform a comparative analysis of the mutational profile identified in ASCC with other squamous cell carcinomas, we analyzed HNSCC and CSCC data sets obtained from cBioPortal (http://www.cbioportal.org/). Briefly, the mutational profiles of the 25 cancer driver genes mutated in ASCC were retrieved from 2 combined cervical cancer data sets (MSK-CESC and TCGA-CESC, *n* = 468) and a head and neck cancer data set (TCGA-HNSC, *n* = 510). Only drivers and putative drivers’ somatic missense or truncating mutations were considered for frequency estimations among cohorts.

### Immunohistochemistry analysis of ASCC.

Immunostaining utilized a Roche Benchmark XT system with anti-CD3 (clone 2GV6, Ventana, Roche), anti-CD8 (clone SP57, Ventana, Roche), anti–PD-L1 (clone SP263, Ventana, Roche), and anti-p16 (clone 6H12, Leica Biosystems) antibodies. Evaluation involved 2 independent pathologists, with discrepancies resolved by a senior pathologist in 4 cases. CD3 and CD8 expression levels were averaged across intra- and peritumoral areas and categorized as low (0%–34%), moderate (35%–64%), or high (65%–100%) based on total tumor-related lymphocyte staining. PD-L1 expression was assessed using the CPS for gastric/gastroesophageal junction adenocarcinoma.

### Statistics.

We used R/Bioconductor for different statistical comparisons outside of MaAsLin’s analysis. To analyze continuous variables, we utilized either 2-tailed *t* tests or Wilcoxon’s tests as appropriate. For categorical data, we employed χ^2^ and Fisher’s tests. Box plots were created in R using the ggplot package. Box plots in figures show the interquartile range, median (line), and minimum and maximum (whiskers). *P* < 0.05 was considered statistically significant.

### Study approval.

This study was approved by the institutional review boards of Fundación Huésped and Hospital de Gastroenterología “Dr. Carlos Bonorino Udaondo,” both in Buenos Aires, Argentina. All participants included in this study gave written informed consent before being involved in the project.

### Data availability.

The raw data have been submitted to NCBI GEO database with accession number GSE253560. Supporting Data Values of figures and Table 1 are available as supplemental files. All codes and scripts used for data preprocessing and analysis are available at the following GitHub repository: https://github.com/mabba777/ASCC-transcriptomics; commit ID d90a47c.

## Author contributions

EL was responsible for investigation, formal analysis, and writing the article. VF, MIF, and PC were responsible for resources and logistics of obtaining samples and clinical data of participants. MES, AMG, JAB, MAP, MG, JR, MK, SI, and SW were responsible for methodology, research assistance, and clinical data of participants. OC and JCR were responsible for resources. MCA conceived the study, supervised, performed formal analysis, and wrote the article.

## Supplementary Material

Supplemental data

Supplemental data set 1

Supplemental data set 2

Supplemental data set 3

Supplemental data set 4

Supplemental data set 5

Supplemental data set 6

Supplemental data set 7

Supplemental data set 8

Supplemental data set 9

Supporting data values

## Figures and Tables

**Table 1:**
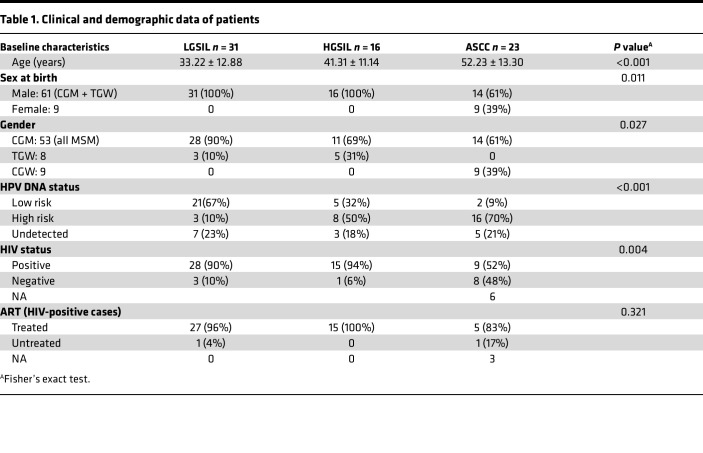
Clinical and demographic data of patients

**Table 2:**
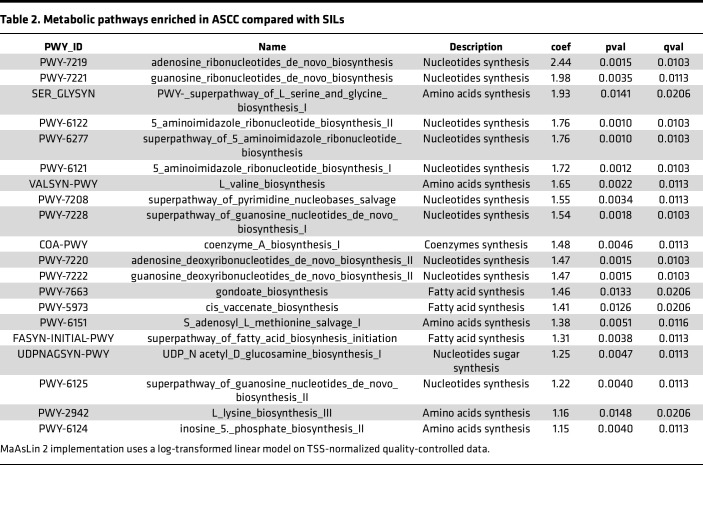
Metabolic pathways enriched in ASCC compared with SILs

## Version 1 07/18/2024

In-Press Preview

## Version 2 08/22/2024

Electronic publication
