# Supplementary material for: Transcriptome and microbiome-immune changes across preinvasive and invasive anal cancer lesions
Source: JCI Insight. 2024 Jul 18;9(16):e180907. doi: 10.1172/jci.insight.180907 (PMC11343604; doi:10.1172/jci.insight.180907)
Supplement: Supplemental data [file jciinsight-9-180907-s034.pdf]

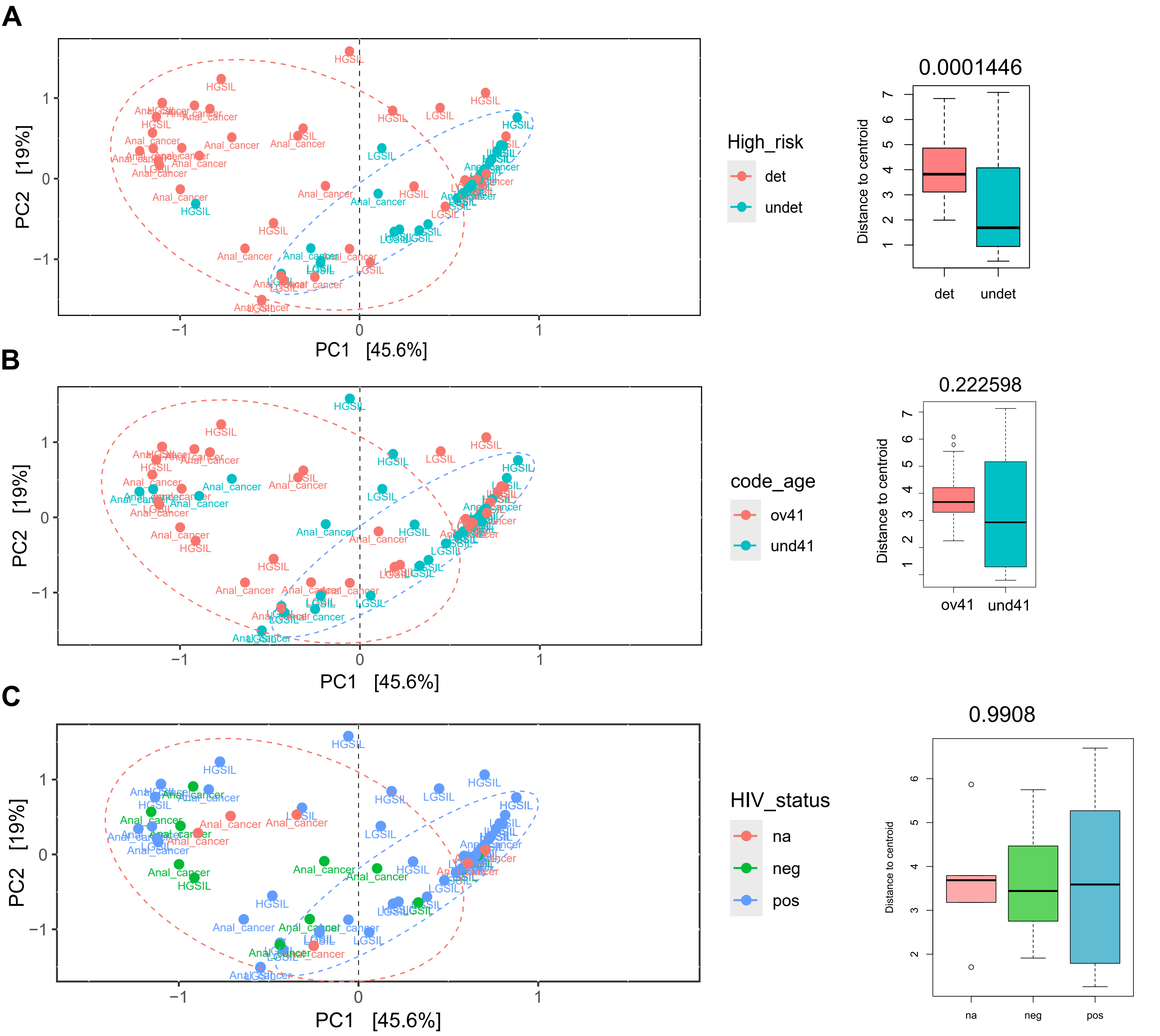

Figure 1: Principal Coordinate Analysis (PCoA) depicting the unsupervised distribution of samples based on microbiota composition (beta diversity). Each panel shows the results of a PERMANOVA analysis for different grouping criteria: **A.** PCoA with samples color coded by HR-HPV status (deteceted vs. undetected). **B.** PCoA with samples color coded by age (ov41: over 41 years-old; und41: under 41 years old) . **C.** PCoA with samples color coded by HIV status (positive vs negative).

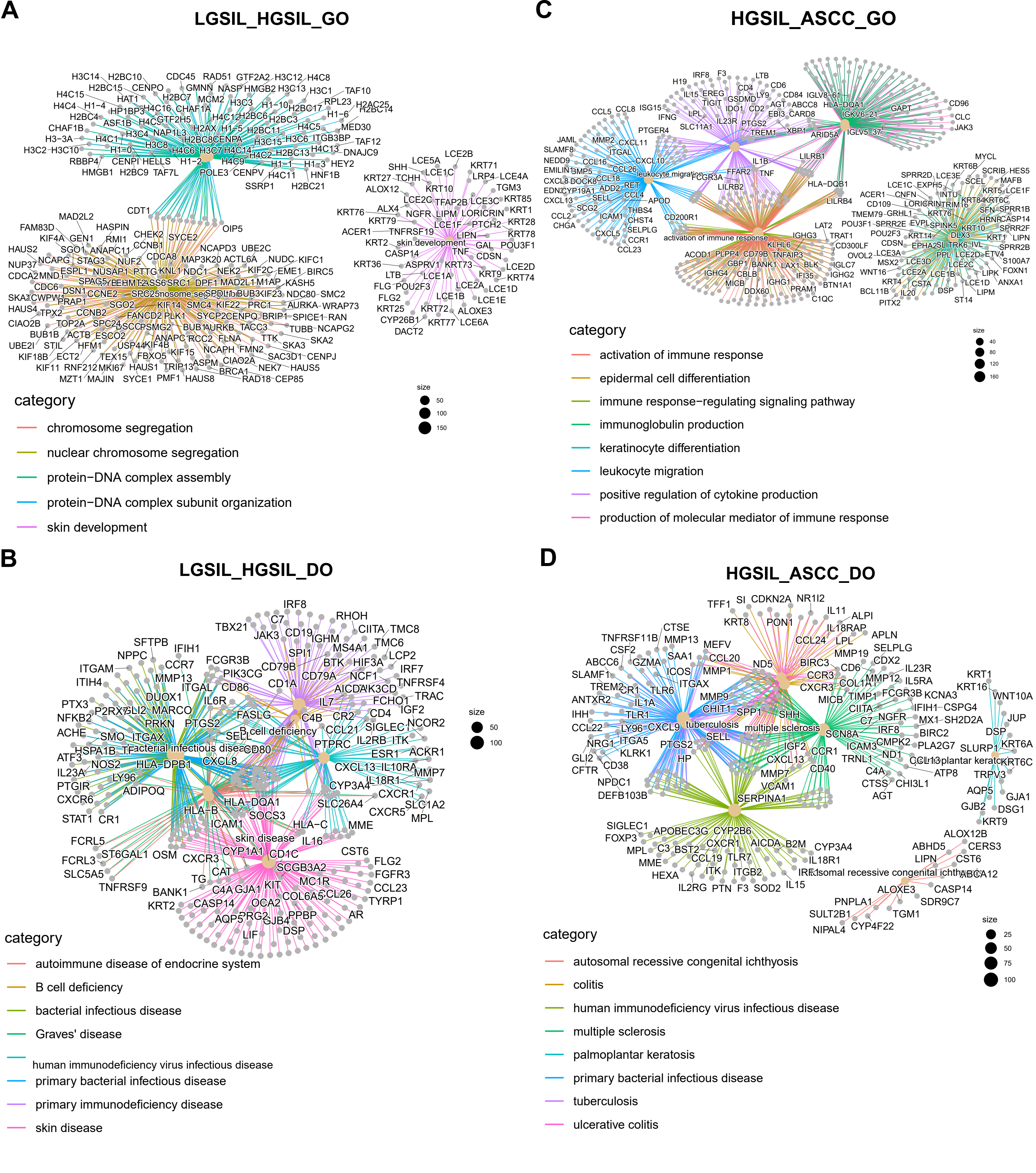

Figure 2: Network Plots depicting relationships between enriched gene sets and significant pathways. **A-B:** Comparison between LGSIL and HGSIL **A.** Gene Ontology (GO) **B.** Disease Ontology (DO) **C-D:** Comparison between HGSIL and ASCC **C.** Gene Ontology (GO) **D.** Disease Ontology (DO)

Members of the University of Miami - Center for AIDS Research/Sylvester Cancer Comprehensive Center Argentina Consortium for Research and Training in Virally Induced AIDS-Malignancies

Dr. Enrique Adrián Mesri

University of Miami - Center for AIDS Research/Sylvester Comprehensive Cancer Center, University of Miami Miller School of Medicine, Miami, Florida, USA.

Dr. Juan Carlos Ramos

University of Miami - Center for AIDS Research/Sylvester Comprehensive Cancer Center, University of Miami Miller School of Medicine, Miami, Florida, USA.

Dr. Sion Williams

University of Miami - Center for AIDS Research/Sylvester Comprehensive Cancer Center, University of Miami Miller School of Medicine, Miami, Florida, USA.

Dr. Omar Coso

Instituto de Fisiología, Biología Molecular y Neurociencias (IFIBYNE-CONICET), Universidad de Buenos Aires, Buenos Aires, Argentina.

Dr. Ana Raimondi

Instituto de Fisiología, Biología Molecular y Neurociencias (IFIBYNE-CONICET), Universidad de Buenos Aires, Buenos Aires, Argentina.

Dr. Julián Naipauer

Instituto de Fisiología, Biología Molecular y Neurociencias (IFIBYNE-CONICET), Universidad de Buenos Aires, Buenos Aires, Argentina.

Dr. Pedro Cahn

Dirección de Investigaciones, Fundación Huésped, Buenos Aires, Argentina.

Dr. Valeria Fink

Dirección de Investigaciones, Fundación Huésped, Buenos Aires, Argentina.

Dr. María Inés Figueroa

Dirección de Investigaciones, Fundación Huésped, Buenos Aires, Argentina.

Dr. Ana M. Gun

Dirección de Investigaciones, Fundación Huésped, Buenos Aires, Argentina.

Dr. Gabriel Adrián Rabinovich

Instituto de Biología y Medicina Experimental (IBYME), Buenos Aires, Argentina.

Dr. Diego Croci

IHEM - Instituto de Histología y Embriología "Dr. Mario H. Burgos" Facultad de Ciencias Médicas de la UNCUYO, Mendoza, Argentina.

Dr. Martin Abba

Centro de Investigaciones Inmunológicas Básicas y Aplicadas (CINIBA), Facultad de Ciencias Médicas, Universidad Nacional de La Plata, La Plata, Argentina.

Dr. Ezequiel Lacunza

Centro de Investigaciones Inmunológicas Básicas y Aplicadas (CINIBA), Facultad de Ciencias Médicas, Universidad Nacional de La Plata, La Plata, Argentina.

Dr. María Eugenia Salas

Centro de Investigaciones Inmunológicas Básicas y Aplicadas (CINIBA), Facultad de Ciencias Médicas, Universidad Nacional de La Plata, La Plata, Argentina.
